# What is the Effect of Changing Running Step Rate on Injury, Performance and Biomechanics? A Systematic Review and Meta-analysis

**DOI:** 10.1186/s40798-022-00504-0

**Published:** 2022-09-04

**Authors:** Laura M. Anderson, Joel F. Martin, Christian J. Barton, Daniel R. Bonanno

**Affiliations:** 1 The Injury Clinic, 100 Fyans Street, South Geelong, VIC 3220 Australia; 2 Discipline of Podiatry, School of Allied Health, Human Services and Sport, La Trobe University, Melbourne, VIC 3086 Australia; 3 La Trobe Sport and Exercise Medicine Research Centre, School of Allied Health, Human Services and Sport, La Trobe University, Melbourne, VIC 3086 Australia; 4 Department of Physiotherapy, School of Primary and Allied Health Care, Faculty of Medicine, Nursing and Health Science, Monash University, Melbourne, VIC 3800 Australia

**Keywords:** Gait retraining, Running retraining, Step rate, Cadence, Running-related injury, Biomechanics, Performance, Systematic review, Meta-analysis

## Abstract

**Background:**

Running-related injuries are prevalent among distance runners. Changing step rate is a commonly used running retraining strategy in the management and prevention of running-related injuries.

**Objective:**

The aims of this review were to synthesise the evidence relating to the effects of changing running step rate on injury, performance and biomechanics.

**Design:**

Systematic review and meta-analysis.

**Data Sources:**

MEDLINE, EMBASE, CINAHL, and SPORTDiscus.

**Results:**

Thirty-seven studies were included that related to injury (*n* = 2), performance (*n* = 5), and biomechanics (*n* = 36). Regarding injury, very limited evidence indicated that increasing running step rate is associated with improvements in pain (4 weeks: standard mean difference (SMD), 95% CI 2.68, 1.52 to 3.83; 12 weeks: 3.62, 2.24 to 4.99) and function (4 weeks: 2.31, 3.39 to 1.24); 12 weeks: 3.42, 4.75 to 2.09) in recreational runners with patellofemoral pain. Regarding performance, very limited evidence indicated that increasing step rate increases perceived exertion ( − 0.49,  − 0.91 to − 0.07) and awkwardness (− 0.72, − 1.38 to − 0.06) and effort (− 0.69, − 1.34, − 0.03); and very limited evidence that an increase in preferred step rate is associated with increased metabolic energy consumption (− 0.84, − 1.57 to − 0.11). Regarding biomechanics, increasing running step rate was associated with strong evidence of reduced peak knee flexion angle (0.66, 0.40 to 0.92); moderate evidence of reduced step length (0.93, 0.49 to 1.37), peak hip adduction (0.40, 0.11 to 0.69), and peak knee extensor moment (0.50, 0.18 to 0.81); moderate evidence of reduced foot strike angle (0.62, 034 to 0.90); limited evidence of reduced braking impulse (0.64, 0.29 to 1.00), peak hip flexion (0.42, 0.10 to 0.75), and peak patellofemoral joint stress (0.56, 0.07 to 1.05); and limited evidence of reduced negative hip (0.55, 0.20 to 0.91) and knee work (0.84, 0.48 to 1.20). Decreasing running step rate was associated with moderate evidence of increased step length (− 0.76, − 1.31 to − 0.21); limited evidence of increased contact time (− 0.95, − 1.49 to − 0.40), braking impulse (− 0.73, − 1.08 to − 0.37), and negative knee work (− 0.88, − 1.25 to − 0.52); and limited evidence of reduced negative ankle work (0.38, 0.03 to 0.73) and negative hip work (0.49, 0.07 to 0.91).

**Conclusion:**

In general, increasing running step rate results in a reduction (or no change), and reducing step rate results in an increase (or no change), to kinetic, kinematic, and loading rate variables at the ankle, knee and hip. At present there is insufficient evidence to conclusively determine the effects of altering running step rate on injury and performance. As most studies included in this review investigated the immediate effects of changing running step rate, the longer-term effects remain largely unknown.

***Prospero Registration*:**

CRD42020167657.

**Key Points**

- Increasing running step rate reduces step length, peak knee flexion angle, peak hip adduction, peak knee extensor moment and foot strike angle
- There is insufficient evidence to determine the effects of changing running step rate on injury or performance
- Increasing running step rate will broadly reduce kinematic and kinetic variables at the ankle, knee and hip

## Background

Running participation provides many health and social benefits [1]. Yet, it is estimated that 50% of runners experience an injury that prevents them from running in a given year, and up to 25% of runners are injured at any given time [2]. Most running-related injuries affect the lower limb and are overuse in nature [2, 3]. The most common injury diagnoses include medial tibial stress syndrome, Achilles tendinopathy, and patellofemoral pain [4].

Many factors are proposed to contribute to running-related injuries including training load, biomechanical factors, and lifestyle and emotional stressors [5]. As running-related injuries often occur following changes to training load [5], it is likely that injuries develop in tissues that are exposed to load that exceeds their capacity [3, 6]. Given the high incidence of running-related injuries, interventions that can decrease tissue loads, assist in maintaining running load, and reduce injury risk without reducing running performance, are likely to be of considerable interest to the running community.

Running retraining (changing running technique) can be used to reduce, or shift tissue loads [7]. Common running retraining strategies include alterations to strike pattern, impact loading, and step rate [8]. A previous mixed-methods study, which synthesised clinical and biomechanical evidence with international expert opinion from coaches and clinicians related to running retraining, found that increasing step rate is the most used strategy in the management of running-related injuries [8]. In addition to considering effects on injury, understanding the relationship between running retraining and performance is needed. Changing a runner’s preferred running gait has been shown by some studies to immediately increase metabolic cost [9, 10], and is therefore proposed to potentially reduce running performance in the short-term. This may not be a major consideration among some recreational runners, but it is likely to be a very important concern among competitive runners. Therefore, it is important for clinicians, coaches, and runners to be aware of the evidence regarding the effects of changing running step rate on measures of performance, in the short- and long-term.

Understanding how changing running step rate affects biomechanics will provide a mechanistic insight into how this retraining strategy could be utilised in managing both injury and performance. A systematic review published in 2012 [11] summarised the immediate effects of changing step rate and stride length in runners from 10 studies, with the review identifying that an increase in step rate decreased centre of mass vertical excursion, ground reaction force, shock attenuation, and energy absorbed at the hip, knee, and ankle joints. Based on these findings, the authors concluded that increasing running step rate may help to reduce the risk of running-related injury [11]. However, the findings of this previous systematic review need to be considered with the knowledge that it did not use meta-analysis to synthesise data, and it focussed on kinematic and kinetic outcomes—performance and injury data were not considered. Additionally, this previous review did not include any studies evaluating step rate as a running retraining intervention over time. There has been a substantial increase in research evaluating the effects of changing running step rate over the past decade and synthesising all contemporary literature through meta-analysis would provide more accurate estimates of these effects.

Therefore, the primary aim of this systematic review and meta-analysis was to synthesise the evidence relating to the effects of altering running step rate on injury and performance. As changing running step rate can affect biomechanics, and therefore tissue loads, a secondary aim of this review was to synthesise the evidence relating to the effects of altering running step rate on spatiotemporal, kinetic, kinematic, muscle function, and impact-related parameters.

## Methods

This systematic review and meta-analysis is reported in accordance with the Preferred Reporting Items for Systematic Reviews and Meta-Analyses (PRISMA) guidelines. The protocol was prospectively registered on the PROSPERO International Prospective Register for Systematic Reviews website in July 2020 (Registration number: CRD42020167657). The review adhered to the protocol without amendments or deviations.

### Literature Search Strategy

Using guidelines provided by the Cochrane Collaboration, a comprehensive search strategy was devised and applied to the following electronic databases with no date restrictions; (i) CINAHL via EBSCO, (ii) EMBASE via OVID, (iii) MEDLINE via OVID and (iv) SPORTDiscus. The first search was performed in April 2020 and repeated in May 2021. The search strategy was deliberately simplified to ensure inclusion of all relevant papers, with all terms searched as free text and keywords (where applicable). Concept 1 covered ‘step rate’ (step frequency OR stride frequency OR step rate OR stride rate OR cadence OR step length OR stride length) AND Concept 2 covered ‘running’ (run* OR jog*). All potential references were imported into Endnote X7 (Thomson Reuters, Carlsbad, California, USA) and duplicates were removed. Two reviewers (LMA and JFM) reviewed all titles returned by the database searches and retrieved suitable abstracts. Where abstracts suggested that papers were potentially suitable, the full-text versions were screened and included in the review if they met the selection criteria. A third reviewer was consulted in case of disagreements (DRB). All studies that met the inclusion criteria had their reference list hand searched. In addition, citation tracking of included studies was performed using Google Scholar.

### Selection Criteria

Studies comparing preferred (i.e. habitual) running step rate to an increase or decrease in step rate, while running were considered for inclusion. A change in running step rate was defined as runners being instructed to alter their preferred step rate by taking more or fewer steps, while running at the same speed. Studies were excluded if step length was manipulated without a corresponding change in step rate, or if participants ran at a set step rate without reporting their preferred step rate, as it was not possible to determine if preferred step rate was altered. Studies were also excluded if other running retraining strategies (e.g. changing foot strike) were used in addition to changing step rate. Case reports and non-English studies were excluded, along with studies with fewer than 10 participants in the cohort or each group [12]. The latter criterion was applied to minimise the risk of potentially false-positive or false-negative findings influencing the evidence synthesis [12].

### Variable Classifications

Injury, performance and biomechanical variables were included in this review. Injury variables included participant-reported measures of pain and/or function. Performance variables were those relating to both physiological measures of performance (e.g. VO_2_) and participant-reported measures of effort (e.g. rate of perceived exertion (RPE)). Biomechanical variables included kinetic, kinematic, and spatiotemporal measures.

### Reported Methodological Quality Assessment

Two independent reviewers (JFM and DRB) rated the quality of included studies using the Downs and Black Quality Index [13]. Any inter-rater discrepancies were resolved by consensus, with a third reviewer (CJB) available if needed. All items were scored as ‘Yes’ (score = 1), ‘No’ (score = 0) or ‘Unclear’ (score = 0), except item 5, which was scored as ‘Yes’ (score = 2), ‘Partial’ (score = 1), ‘No’ (score = 0) or ‘Unclear’ (score = 0). Based on assessment scores, studies were categorised as high quality (≥ 20 out of maximum possible score 28), moderate quality (17–19) or low quality (≤ 16) [12]. The Downs and Black Quality Index has been shown to have high internal consistency, test–retest and inter-rater reliability, and high criterion validity [13].

### Data Management

All study data were extracted from included studies by the primary author (LMA) and double-checked by a second author (JFM). If sufficient data were not reported in the published article or related supplementary material, corresponding authors were contacted via email to request further data. If additional data were not provided, the best available data from the published article were still included in the review.

#### Statistical Analysis

Means and standard deviations were used to calculate the standardised mean difference (SMD) with 95% confidence intervals (CI) for variables of interest. Data were pooled where possible. Meta-analysis was performed using the Cochrane Collaboration Review Manager 5.4 software. A random-effects model was used for the meta-analyses due to differences between the study design, interventions, participants, and research settings.

#### Data Synthesis

Levels of evidence were determined using a modified version of the van Tulder criteria [14]: (i) strong evidence provided by consistent findings among multiple studies, including at least three high-quality studies; (ii) moderate evidence provided by consistent findings among multiple studies, including at least three moderate- or high-quality studies or two high-quality studies; (iii) limited evidence provided by consistent findings among multiple low- or moderate-quality studies, or one high-quality study; (iv) very limited evidence provided by findings from one low or moderate quality study; and, (v) conflicting evidence provided by inconsistent findings among multiple studies, regardless of quality.

Definition of consistent findings (i.e. statistical homogeneity) was based on an *I*^*2*^ of 50% or less. *I*^*2*^ values greater than 50% were classified as inconsistent (i.e. statistical heterogeneity), with level of evidence downgraded one level if pooled results were significant. Calculated SMD magnitudes were classified as small (≤ 0.59), medium (0.60–1.19), or large (≥ 1.20) [12].

## Results

### Search Strategy and Reported Quality

**Fig. 1:**
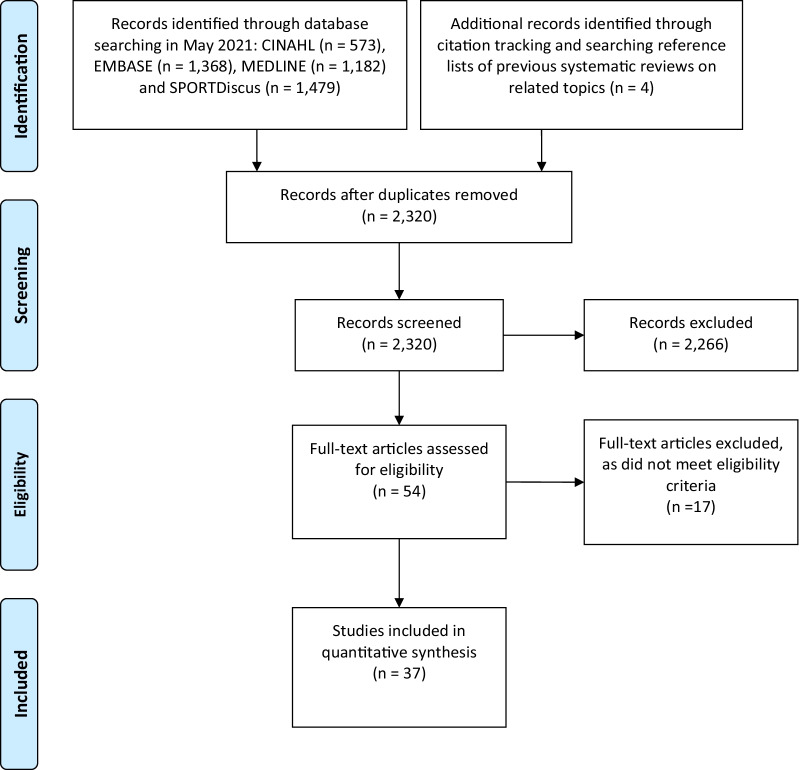
PRISMA flow diagram for the selection of studies.

The initial search identified 4602 titles. Following removal of duplicate publications, titles of 2320 publications were evaluated. The full text of 54 articles were retrieved, and 37 studies were identified for inclusion (see Fig. 1). Thirty-three studies investigated the immediate effects of changing step rate on performance and biomechanics, and four studies evaluated the longer-term effects of changing step rate on injury and biomechanics. The primary reasons for exclusion of studies were combined running retraining strategies [15–18], and manipulation of step length with no change in step rate [19–21]. In addition to data being extracted directly from the 37 included studies where possible, additional data were provided by 5 authors upon request [22–26].

Characteristics of the 37 included studies are given in Table 1. The results of the Downs and Black Quality Index scores for each study are shown in Table 2. Of the 37 included studies, 17 were high quality [22, 27–42], 19 were moderate quality [23, 25, 26, 43–58], and 1 was low quality [24].

**Table 1:** Characteristics of included studies

Study	Study design and setting	Sample	Comparison	Outcome measures
Adams et al. [43]	Cross-sectional Setting: laboratory, instrumented treadmill Step rate cue: verbal (“increase the number of times your foot hits the ground by 10%”) Speed: self-selected (comfortable) Footwear: not described Additional: running watch used for data collection	20 recreational runners (running experience, 11.5 ± 6.9 years; average running distance, 37.3 ± 27.8 km/week)	Preferred step rate versus “high cadence”	Kinetics: Peak vertical GRF Braking impulse Average vertical loading rate Instantaneous vertical loading rate Gait: Step rate Vertical oscillation Ground contact time
Allen et al. [22]	Cross-sectional Setting: laboratory, treadmill Step rate cue: audible and visual metronome Speed: self-selected (moderate intensity) Footwear: participant’s own running shoes	40 recreational runners (rearfoot strike pattern) (age, 36.0 ± 9.1 years; average running distance, 24.9 ± 20.9 mi/week; male, 17; female, 23)	Preferred step rate versus + 5%, + 10%, + 15%	Gait: Foot strike pattern Foot inclination Step rate
Baggaley et al. [44]	Cross-sectional Setting: laboratory, instrumented treadmill Step rate cue: audible metronome Speed: predetermined (3.33 m/s) Footwear: standardised (Salomen X-Scream 3d)	19 recreational runners 10 females (age, 27 ± 10 years; mass, 66.8 ± 6.9 kg), 9 males (age, 28 ± 8 years; mass, 73.7 ± 8.0 kg)	Preferred step rate versus − 10%, + 10%,	Kinetics: Peak sacral acceleration Peak tibial acceleration Negative hip work Negative knee work Negative ankle work Impact attenuation Gait: Step length
Baumgartner et al. [27]	Randomised controlled trial Setting: laboratory, treadmill (baseline and at 6-week follow-up); overground or treadmill (retraining period) Step rate cue: visual feedback (wristwatch) Speed: self-selected Footwear: participant’s own running shoes	38 recreational runners. 20 experimental (age, 37.7 ± 9.8 years; mass, 80.5 ± 16.6 kg); 18 controls (age, 39.7 ± 14.8 years; mass, 71.6 ± 12.7 kg)	Experimental: preferred step rate + 10% Control: preferred step rate	Gait: Stride rate Note: data collected at baseline and 6-weeks
Bonacci et al. [29]	Cross-sectional Setting: laboratory, instrumented treadmill Step rate cue: audible metronome Speed: not described Footwear: standardised (control shoe: Asics Gel Cumulus 16; minimalist shoe: Vibram Seeya)	15 recreational runners (age, 32.6 ± 9.6 years; mass, 68.9 ± 11.0 kg; average running distance, 15.6 ± 7.4 km/week; female, 12; male, 3) Clinical diagnosis of patellofemoral pain	Preferred step rate versus + 10%	Kinematics: Peak knee flexion angle Kinetics: Peak knee extensor moment Peak patellofemoral joint stress Peak patellofemoral joint reaction force
Bonacci et al. [28]	Cross-sectional Setting: laboratory, instrumented treadmill Step rate cue: audible metronome Speed: not described Footwear: standardised (control shoe: Asics Gel Cumulus 16; minimalist shoe: Vibram Seeya)	15 recreational runners (rearfoot strike pattern) (age, 32.6 ± 9.6 years; mass, 68.9 ± 11.0 kg; average running distance, 15.6 ± 7.4 km/week; female, 12; male, 3) Clinical diagnosis of patellofemoral pain	Preferred step rate versus + 10%	Approximate entropy: Hip: flexion/extension, internal rotation/external rotation Knee: flexion/extension, adduction/abduction, internal rotation/external rotation Ankle: dorsiflexion/plantarflexion, inversion/eversion, internal rotation/external rotation
Bowerstock et al. [30]	Cross-sectional Setting: laboratory, instrumented treadmill Step rate cue: audible metronome Speed: self-selected (equivalent to speed of a 30 min training run) Footwear: standardised (Saucony Progrid Ride)	19 recreational runners (rearfoot strike pattern) 10 females (age 22.7 ± 2.5 years; mass, 57.8 ± 7.0 kg); 9 males (age, 22.5 ± 3.1 years; mass, 79.9 ± 7.5 kg)	Preferred step rate versus − 10%, + 10% (RFS) Preferred step rate versus − 10%, + 10% (FFS)	Kinetics: Vertical GRF Tibiofemoral joint contact force Peak force Braking GRF Hamstring Peak force Quadriceps peak force Gastrocnemius peak force Hamstring impulse Hamstring impulse/km Quadriceps impulse Quadriceps impulse/km Gastrocnemius impulse Gastrocnemius impulse/km Gait: Step length Stance time
Bramah et al. [23]	Case series Setting: laboratory, treadmill (baseline); overground or treadmill participant selected (retraining period) Gait retraining programme: 4 weeks Step rate cue: audible metronome (weeks 1–2) and self-monitored using GPS smartwatch (weeks 3–4) Speed: self-selected (preferred) Footwear: participant’s own running shoes	12 runners with patellofemoral pain (age, 39.9 ± 6.5 years; mass, 61.0 ± 6.5 kg; female, 8; male, 4)	Preferred step rate versus + 10%	Clinical: Worst pain (NRS) Lower Extremity Functional Scale Self-reported longest distance run pain-free Total weekly running volume Kinematics: Peak contralateral pelvic drop Peak hip adduction Peak hip internal rotation Peak knee flexion Gait: Stride rate Note: Data collected at baseline, 4-weeks, and 3-months
Busa et al. [31]	Cross-sectional Setting: laboratory, instrumented treadmill Step rate cue: audible metronome Speed: self-selected (preferred) Footwear: standardised (T7 Brooks)	12 recreational runners (age, 29. 7 ± 4.4 years; mass, 72.1 ± 13.9 kg; female, 4; male, 8)	Preferred step rate versus − 20%, − 10%, + 10%, + 20%	Kinetics: Tibial impact acceleration peak Head impact acceleration peak Head active acceleration peak Tibial signal power magnitude Tibial signal power magnitude Head signal power magnitude Head signal power magnitude Shock attenuation active phase magnitude Shock attenuation impact phase magnitude Gait: Step rate
Chumanov et al. [32]	Cross-sectional Setting: laboratory, instrumented treadmill Step rate cue: audible metronome Speed: self-selected (preferred) Footwear: not described	45 recreational runners (age, 32.7 ± 15.5 years; mass, 69.5 ± 13.1 kg; female, 20; male, 25; average running distance, 29.8 ± 15.5 mi/wk)	Preferred step rate versus + 5%, + 10%	Electromyography (stance phase: 0–15% GC; 30–50% GC and swing phase: 70–80% GC; 80–90% GC; 90–100% GC): Vastus lateralis Rectus femoris Tibialis anterior Medial gastrocnemius Lateral hamstring Medial hamstring Gluteus maximus Gluteus medius
Clarke et al. [45]	Cross-sectional Setting: laboratory, treadmill Step rate cue: audible metronome Speed: predetermined (3.8 m/s) Footwear: not described	10 recreational runners, average running distance (25–135 km/week)	Preferred step rate versus − 10%, − 5%, + 5%, + 10%	Kinematics: Hip (IC) Knee (IC), max after (IC) Ankle (IC) Horizontal foot velocity (IC) Vertical foot velocity (IC) Kinetics: Peak shank deceleration Gait: Step rate Stride length Relative stride Length Flight time Support time
Connick and Li [46]	Cross-sectional Setting: laboratory, standard treadmill Step rate cue: audible metronome Speed: predetermined (13 km/h) Footwear: not described	11 trained runners (10 km PB 34.8 min ± 3.1) (age, 26.4 ± 7.1 years; mass 68.5 ± 8.3 kg; male, 11)	Preferred step rate versus − 8%, − 4%, + 4%, + 8%	Electromyography: Bicep femoris Vastus lateralis Gastrocnemius
Dewolf and De Jaeger [24]	Cross-sectional Setting: laboratory, instrumented treadmill Step rate cue: audible metronome Speed: predetermined speed (12 and 14 km/h) Footwear: not described	20 runners (age, 22.1 ± 2.2 years; mass, 72.6 ± 11 kg; male, 15; female, 5)	Preferred step rate versus − 10% (14 km/h)	
dos Santos et al. [34]	Cross-sectional Setting: laboratory, instrumented treadmill Step rate cue: audible metronome Speed: self-selected (comfortable) Footwear: standardised (Asics Gel-Eq. 5)	31 recreational runners (rearfoot strike pattern) (age, 27.7 ± 5.4 years; mass 72.1 ± 0.1 kgs; average running distance, 35.7 ± 18.3 km/week; female, 11; male, 20)	Preferred step rate versus + 10%	Kinematics (IC, average stance, peak stance): Trunk: flexion Hip: internal rotation, adduction, abduction, flexion Knee: external rotation, adduction, abduction, flexion Ankle: plantarflexion, dorsiflexion
dos Santos et al. [33]	Cross-sectional Setting: laboratory, instrumented treadmill Step rate cue: audible metronome Speed: self-selected (preferred) Footwear: not described	19 recreational runners (rearfoot strike pattern) (age, 28.1 ± 5.0 years; average running distance, 26.6 ± 8.9 km/week; female, 11; male, 8)	Preferred step rate versus + 10%	Kinematics: Foot strike angle Trunk flexion angle Peak knee flexion during stance phase Kinetics: Peak GRF Peak patellofemoral joint stress Patellofemoral joint stress-time integral Hip extensor moment (SP) Plantarflexion moment (SP) Peak knee extensor moment (SP) Gait: Step length Step rate Number of steps per km
Garofolini et al. [58]	Cross-sectional Setting: laboratory, instrumented treadmill Step rate cue: real-time biofeedback; vertical bar graph Speed: 11 km/h Footwear: standardised (neutral shoe provided)	20 active males (age, 28.1 ± 2.8 years; mass, 75.8 ± 5.7 kgs)	Preferred step rate versus + 10 to + 15%	Kinetics: Loading rate Gait: Step rate Foot strike angle
Gerrard and Bonanno [35]	Cross-sectional Setting: laboratory, instrumented treadmill Step rate cue: audible and visual metronome Speed: self-selected (equivalent to 20 min of moderate intensity) Footwear: participant’s own running shoes	32 recreational runners (age, 28.2 ± 8.0 years; mass, 67.5 ± 13.8 kg; average running distance 30.4 ± 2 4.4 km/week; female, 16; male, 16)	Preferred step rate versus − 10%, − 5%, + 5%, + 10%	Kinetics: Max force Peak pressure Contact area Gait: Contact time
Hafer et al. [47]	Cross-sectional Setting: laboratory, overground (30 m runway) Step rate cue: audible metronome Speed: self-selected (comfortable) Footwear: standardised (New Balance 1062)	10 recreational runners (rearfoot strike pattern) (age, 32.7 ± 7.5 years; mass, 63.9 ± 7.0 kg; female, 8; male, 2)	Preferred step rate versus + 10%	Kinematics: Excursion (ROM during stance phase) / angle (peak ROM) / time (% of gait cycle when peak angle occurred) Knee: flexion, internal rotation Shank: internal rotation Rearfoot: eversion Segment coordination (terminal swing; early stance; mid stance; late stance) Sagittal thigh rotation versus sagittal shank rotation Sagittal thigh rotation versus transverse shank rotation Transverse thigh rotation versus transverse shank rotation Transverse shank rotation versus frontal rearfoot rotation Coordination variability (terminal swing; early stance; mid stance; late stance) Sagittal thigh rotation versus sagittal shank rotation Sagittal thigh rotation versus transverse shank rotation Transverse thigh rotation versus transverse shank rotation Transverse shank rotation versus frontal rearfoot rotation
Halvorsen et al. [48]	Cross-sectional Setting: laboratory, treadmill Step rate cue: visual and audible feedback for initial instructions, and audible feedback only for running trials Speed: set speed 16 km/h (12-14 km/h for familiarisation and warm-up) Footwear: not described	16 national level competitive runners, triathletes and orienteers (age, 28 ± 5 years; mass, 71.7 ± 5.7 kg; male, 16)	Preferred step rate versus − 10%, − 5%	Performance VO_2_ Blood lactate RPE (BORG scale)
Heiderscheit et al. [49]	Cross-sectional Setting: laboratory, instrumented treadmill Step rate cue: audible metronome Speed: self-selected (equivalent to moderate intensity run) Footwear: not described	45 recreational runners (age, 32.7 ± 15.5 years; mass, 69.5 ± 13.1 kg; average running distance 29.8 ± 15.5 km/week; female, 20; male, 25)	Preferred step rate versus − 10%, − 5%, + 5%, + 10%	Kinematics: Hip: peak flexion angle, peak adduction angle, peak internal rotation angle Knee: IC flexion angle, peak flexion angle Ankle: IC foot inclination COM vertical excursion Kinetics: Hip: IC extension moment and peak abduction moment, peak internal rotation moment, negative and positive work Knee: peak extension moment, negative and positive work Peak vertical GRF Braking impulse Impact transient occurrence Gait: Step length Stance duration Initial contact COM – heel distance Additional: RPE
Hobara et al. [25]	Cross-sectional Setting: laboratory, instrumented treadmill Step rate cue: audible metronome Speed: predetermined (2.5 m/s) Footwear: not described	10 recreational runners (age, 28.8 ± 3.0 years; mass, 71.5 ± 9.3 kg; male, 10)	Preferred step rate versus − 30%, − 15%, + 15%, + 30%	Kinetics: Vertical GRF Vertical average loading rate Vertical impact peak Vertical instantaneous loading rate
Huang et al. [50]	Cross-sectional Setting: laboratory, instrumented treadmill Step rate cue: audible metronome Speed: self-selected Footwear: participant’s own running shoes	19 recreational runners (15 midfoot strike pattern, 4 rearfoot strike pattern) (age, 21.7 ± 2.6 years; mass, 68.5 ± 6.3 kg; male, 19)	Preferred step rate versus + 10%	Kinetics: Peak tibial acceleration Vertical impact peak GRF Vertical average loading rate Vertical instantaneous loading rate Additional: Awkwardness (VAS) Effort (VAS)
Hunter and Smith [42]	Cross-sectional Setting: laboratory; instrumented treadmill Step rate cue: computer-based metronome Speed: individual 60 min maximal run pace Footwear: not described	16 recreational runners (age, 28 ± 8 years; mass, 70.4 ± 10.5 kg; female, 5; male, 11)	Preferred step rate versus − 4%, − 8%, + 4%, + 8%	VO_2_ Stride frequency Stiffness
Lenhart et al. [51]	Cross-sectional Setting: laboratory, instrumented treadmill Step rate cue: audible metronome Speed: self-selected (preferred) Footwear: not described	30 recreational runners (age, 33 ± 14 years; mass, 68.6 ± 10.9 kg; female, 15; male, 15)	Preferred step rate versus − 10%, + 10%	Data not provided Kinematics: Knee flexion angle Peak knee flexion angle, knee flexion at IC Kinetics: Patellofemoral force Peak patellofemoral force Patellofemoral stance phase loading rate Peak patellofemoral loading rate Peak vertical GRF Additional: Peak muscle force Vastus lateralis Rectus femoris Soleus Patellar tendon Tibialis anterior Biceps femoris Semimembranosus Medial gastrocnemius (0–40%; 80–99%) Gluteus medius (0–40%; 80–99%) Gluteus maximus (0–40%; 80–99%) *0–40%* = *late stance / early swing* *80–99%* = *late swing*
Lenhart et al. [36]	Cross-sectional Setting: laboratory, instrumented treadmill Step rate cue: audible metronome Speed: self-selected (preferred) Footwear: not described	30 recreational runners (age, 33 ± 14 years; mass, 68.6 ± 10.9 kg; female, 15; male, 15)	Preferred step rate versus − 10%, + 10%	Kinetics: *Positive and Negative Work* Biceps femoris long head Semimembranosus Gluteus maximus Gluteus medius Gluteus minimus Tensor fasciae latae Rectus femoris Sartorius Psoas Iliacus Adductor magnus Adductor brevis Adductor longus Piriformis Performance Peak muscle forces *Stance and late swing* Biceps femoris long head Semimembranosus Gluteus maximus Gluteus medius *Stance and early swing and late swing* Glutes minimus Piriformis *Early swing* Tensor fasciae latae Sartorius Psoas Iliacus Adductor brevis Adductor longus *Stance* Adductor magnus *Stance and early swing* Rectus femoris
Lenhart et al. [52]	Cross-sectional Setting: laboratory, instrumented treadmill Step rate cue: audible metronome speed: self-selected (preferred) Footwear: not described	22 recreational runners (mass, 71.0 ± 8.8 kg; average running distance 45.5 ± 24.1 km/week; female, 7; male, 15)	Preferred step rate versus − 10%, + 10%	Kinetics: Patellofemoral joint contact force Patellofemoral joint contact area loading rate Patellofemoral joint contact pressure loading rate
Lieberman et al. [37]	Cross-sectional Setting: laboratory, instrumented treadmill Step rate cue: audible metronome Speed: predetermined (3.0 m/s) Footwear: not described	14 recreational runners (mass, 72.9 ± 11.6 kg; female, 2; male, 12)	Step rates: 75, 80, 85, 90, 95 strides/min	Kinematics: Max hip flexion Landing position of foot relative to hip Landing position of foot relative to knee Kinetics: Impact peak GRF Braking impulse Vertical loading rate Maximum hip flexion moment Additional: Cost of transport
Mercer et al. [53]	Cross-sectional Setting: laboratory, treadmill Step rate cue: audible metronome Speed: 3 set running speeds: 3.13 m/s, 3.58 m/s, 4.02 m/s Footwear: not described	10 well-trained runners (age, 23.0 ± 5.0 years; mass, 66.3 ± 8.8 kg; female, 4; male, 6)	Preferred step rate versus − 15%, + 15% (at 3 different pre − determined running speeds)	VO_2_
Morin et al. [54]	Cross-sectional Setting: laboratory, instrumented treadmill Step rate cue: audible metronome Speed: predetermined (3.33 m/s) Footwear: not described	10 recreational runners (age, 28.6 ± 6.4 years; mass, 75.6 ± 10.4 kg; male, 10)	Preferred step rate versus − 30%, + 30%	Kinematics: Downward displacement of COM Kinetics: Vertical GRF (max) Leg compression Vertical stiffness Leg stiffness Gait: Contact time Aerial time Duty factor
Neal et al. [55]	Case series Setting: laboratory, instrumented treadmill Gait retraining programme: 18 sessions over 6 weeks Step rate cue: audible metronome (faded feedback) used in sessions 1–12. No feedback provided in sessions 13–18 Speed: self-selected (preferred) Footwear: participant’s own running shoes	10 runners with patellofemoral pain (age, 31.6 ± 5.5 years; mass, 67.7 ± 9.8 kg; average running distance, 17.0 ± 9.8 km/week; female, 6; male, 4)	Preferred step rate versus + 7.5%	Clinical: Average pain (NRS) Worst pain (NRS) Kujala Scale (function) Kinematics: Peak contralateral pelvic drop Peak hip adduction Peak hip internal rotation Peak hip flexion Peak knee flexion Electromyography: Gluteus maximus Gluteus medius Semitendinosus Vastus medialis oblique Gait: Step rate Note: Data collected at baseline and 6-weeks. Biomechanical data not included in this review as only available for *n* = 9
Quinn et al. [56]	Case–control study Setting: laboratory, treadmill Step rate cue: audible metronome Speed: 3.4 to 3.8 m/s Footwear: participant’s own running shoes	22 well-trained female runners (step rate < 176 steps/min) 11 experimental (age, 22.9 ± 5.0 years; mass, 58.1 ± 8.3 kg; 5 k PB 19.8 ± 1.4 min); 11 controls (age, 21.3 ± 1.4 years; mass, 58.0 ± 2.5 kg; 5 k PB 19.9 ± 1.6 min)	Preferred step rate versus 180 steps/minute	Gait: Step frequency Step length Additional: RE VO_2_ max Ventilation Heart rate Note: Data collected at baseline and 12-days
Swinnen et al. [26]	Cross-sectional Setting: laboratory, instrumented treadmill Step rate cue: audible metronome Speed: self-selected and 12kmp/h Footwear: not described	17 experienced runners (age, 23.7 ± 3.8 years; mass, 69.1 ± 7.7 kg; female, 4; male, 13)	Preferred step rate versus − 15%, − 8%, + 8%, + 15%	Kinetics: Average positive ankle power Average positive knee power Average positive hip power Gait: Stride frequency Step length Ground contact time Duty factor Additional: Metabolic energy consumption Muscle activation
Wang et al. [38]	Randomised controlled trial Setting: laboratory, treadmill and 10 m runway (baseline testing); overground (retraining programme) Step rate cue: audible metronome Speed: 3.33 m/s (baseline testing); self-selected (retraining programme) Footwear: standardised (Nike Pegasus 34)	30 recreational male runners (rearfoot strike pattern) 12 experimental (age, 23.6 ± 7.5 years; mass, 71.8 ± 4.9 kg); 12 controls (age, 23.7 ± 1.2 years; mass, 70.8 ± 7.3 kg)	Preferred step rate versus + 7.5%	Kinematics: Time from IC contact to impact peak Foot angle at IC Max dorsiflexion during stance Max knee flexion during stance Max hip flexion during stance Vertical excursion of Centre of gravity Vertical velocity of centre of gravity at IC Kinetics: Vertical instantaneous load rate Vertical average load rate Lower extremity stiffness Impact peak (BW) Gait: Step rate Step length Note: Data collected at baseline and 12-weeks
Wellenkotter et al. [39]	Cross-sectional Setting: laboratory, treadmill Step rate cue: audible and visual metronome Speed: self-selected (preferred) Footwear: standardised (New Balance 625)	38 recreational runners (age, 23.0 ± 3.5 years; mass, 30.6 ± 4.7 kg; female, 19; male, 19)	Preferred step rate versus − 5%, + 5%	Kinetics: Total foot plantar loading Heel plantar loading Medial metatarsal plantar loading Central metatarsal plantar loading Lateral metatarsal plantar loading Peak force Force time integral Peak pressure Pressure time integral Gait: Contact time
Willy et al. [40]	Randomised control trial Setting: laboratory, instrumented treadmill (baseline testing) Overground or treadmill (retraining programme) Step rate cue: real-time visual feedback (wristwatch) on runs 1–3, 5 and 7 (no feedback on runs 4, 6 and 8) Speed: self-selected Footwear: not described	30 recreational runners 16 experimental (age, 251.9 ± 16.3 months; BMI, 23.0 ± 2.6 kg/m^2^; female 9, male 7); 14 controls (age, 248.8 ± 15 months; BMI, 23.4 ± 3.3 kg/m^2^; female 9, male 7)	Preferred step rate versus + 7.5%	Kinematics: Peak hip Adduction Kinetics: Vertical ground reaction force Instantaneous vertical load rate Average vertical load rate Eccentric knee work per stance Eccentric knee work per km Knee joint power Gait: Steps per minute Note: Data collected at baseline, post retraining period (after 8 runs) and 1-month
Yong et al. [41]	Cross-sectional Setting: laboratory, overground 16.5 m Step rate cue: audible metronome Speed: self-selected Footwear: standardised (Saucony Ride 7) Additional: participants completed a practice of intervention on a treadmill prior to overground testing	17 recreational runners (rearfoot strike pattern) (age, 32.1 ± 9.8 years; mass, 64.9 ± 12.5 kg; female, 11; male, 6)	Preferred step rate versus + 10%	Kinematics: Peak hip adduction angle Kinetics: Loading Rate Peak tibial acceleration Peak absolute free moment
Zimmerman et al. [57]	Cross-sectional Setting: laboratory, instrumented treadmill Step rate cue: audible metronome Speed: predetermined (10 km/h) Footwear: participant’s own running shoes	12 recreational runners Clinical diagnosis of exercise related leg pain	Step rate = 180	Kinetics: Heel max GRF Midfoot max GRF Forefoot max GRF Heel max pressure midfoot max pressure forefoot max pressure Gait: Stride length Step rate

*GRF* ground reaction force, *BW* body weight, *IC* initial contact, *SP* stance phase, *COM* centre of mass, *VAS* visual analogue scale, *NRS* numerical rating scale, *PB* personal best

**Table 2:** Downs and Black Quality Index results for each study

	1. Clear aim/ hypothesis	2. Outcome measures described	3.Patient characteristics described	4. Intervention clearly described	5. Principal confounders	6. Findings clearly described	7. Random variability	8. Adverse events	9. Lost to follow-up	10. Probability values reported	11. Asked subjects representative	12. Inc. subjects representative	13. Staff & facilities representative	14. Attempt blinding participants
Allen et al. [22]	1	1	1	1	2	1	1	0	1	1	U	U	1	1
Willy et al. [40]	1	1	1	1	2	1	1	0	1	1	U	U	0	1
Baumgartner et al. [27]	1	1	1	1	2	1	1	0	1	1	U	U	1	0
Bowerstock et al. [30]	1	1	1	1	2	1	1	0	1	1	U	U	0	0
Gerrard and Bonanno [35]	1	1	1	1	2	1	1	0	1	1	U	U	0	1
Bonacci et al. [29]	1	1	1	1	2	1	1	0	1	1	U	U	0	0
Bonacci et al. [28]	1	1	1	1	2	1	1	0	1	1	U	U	0	0
Busa et al. [31]	1	1	1	1	2	1	1	0	1	1	U	U	0	0
Chumanov et al. [32]	1	1	1	1	2	1	1	0	1	1	U	U	0	0
dos Santos et al. [34]	1	1	1	1	2	1	1	0	1	1	U	U	0	0
dos Santos et al. [33]	1	1	1	1	2	1	1	0	1	1	U	U	0	0
Hunter and Smith [42]	1	1	1	1	2	1	1	0	1	1	U	U	0	0
Lenhart et al. [36]	1	1	1	1	2	1	1	0	1	1	U	U	0	0
Lieberman et al. [37]	1	1	1	1	2	1	1	0	1	1	U	U	0	0
Wang et al. [38]	1	1	1	1	2	1	1	0	1	1	U	U	0	0
Wellenkotter et al. [39]	1	1	1	1	2	1	1	0	1	1	U	U	0	0
Yong et al. [41]	1	1	1	1	2	1	1	0	1	1	U	U	0	0
Baggaley et al. [44]	1	1	1	1	2	1	1	0	1	1	U	U	0	0
Hafer et al. [47]	1	1	1	1	2	1	1	0	1	1	U	U	0	0
Heiderscheit et al. [49]	1	1	1	1	2	1	1	0	1	0	U	U	0	0
Huang et al. [50]	1	1	1	1	2	1	1	0	1	0	U	U	0	0
Lenhart 2014 et al. [51]	1	1	1	1	2	1	1	0	1	0	U	U	0	0
Lenhart et al. [52]	1	1	1	1	2	1	1	0	1	0	U	U	0	0
Neal et al. [55]	1	1	1	1	2	1	1	0	1	1	U	U	0	0
Swinnen et al. [26]	1	1	1	1	2	0	1	0	1	1	U	U	0	0
Zimmerman et al. [57]	1	1	1	1	1	1	1	0	1	0	1	U	0	0
Adams et al. [43]	1	1	1	1	1	1	1	0	1	1	U	U	0	0
Bramah et al. [23]	1	1	1	1	2	1	1	0	1	0	U	U	0	0
Garofolini et al. [58]	1	1	1	1	2	1	0	0	1	1	U	U	0	0
Halvorsen et al. [48]	1	1	1	1	1	1	1	0	1	1	U	U	0	0
Hobara et al. [25]	1	1	1	1	2	0	1	0	1	0	U	U	0	0
Mercer et al. [53]	1	1	1	1	1	1	1	0	1	1	U	U	0	0
Morin et al. [54]	1	1	1	1	1	1	1	0	1	0	U	U	0	0
Quinn et al. [56]	1	1	1	1	1	1	1	0	1	1	U	U	0	0
Clarke et al. [45]	1	1	0	1	1	1	1	0	1	0	U	U	0	0
Connick and Li [46]	1	1	1	1	1	1	0	0	1	0	U	U	0	0
Dewolf and De Jaeger [24]	1	1	1	1	1	1	1	0	1	0	U	U	0	0

	15. Attempt blinding clinicians	16. Data dredging	17. Analysis adjust for follow-up	18. Statistics appropriate	19. Compliance with intervention	20. Outcome measures accurate	21. Recruited same population	22. Recruited over same period	23. Randomised to groups	24. Randomisation concealed	25. Adjustment for confounding	26. Accounted for lost to follow-up	27. Power to detect clinical effect	Total (score out of 28)
Allen et al. [22]	0	1	1	1	1	0	U	1	1	1	1	1	1	22
Willy et al. [40]	U	1	1	1	1	1	U	1	1	1	1	1	1	22
Baumgartner et al. [27]	0	1	1	1	1	1	U	1	1	U	1	1	1	21
Bowerstock et al. [30]	0	1	1	1	1	1	U	1	1	1	1	1	1	21
Gerrard and Bonanno [35]	0	1	1	1	1	1	U	1	1	U	1	1	1	21
Bonacci et al. [29]	0	1	1	1	1	1	U	1	1	U	1	1	1	20
Bonacci et al. [28]	0	1	1	1	1	1	U	1	1	U	1	1	1	20
Busa et al. [31]	0	1	1	1	1	1	U	1	1	U	1	1	1	20
Chumanov et al. [32]	0	1	1	1	1	1	U	1	1	U	1	1	1	20
dos Santos et al. [34]	0	1	1	1	1	1	U	1	1	U	1	1	1	20
dos Santos et al. [33]	0	1	1	1	1	1	U	1	1	U	1	1	1	20
Hunter and Smith [42]	0	1	1	1	1	1	U	1	1	U	1	1	1	20
Lenhart et al. [36]	0	1	1	1	1	1	U	1	1	U	1	1	1	20
Lieberman et al. [37]	0	1	1	1	1	1	U	1	1	U	1	1	1	20
Wang et al. [38]	0	1	1	1	1	1	U	1	1	0	1	1	1	20
Wellenkotter et al. [39]	0	1	1	1	1	1	U	1	1	U	1	1	1	20
Yong et al. [41]	0	1	1	1	1	1	U	1	1	U	1	1	1	20
Baggaley et al. [44]	0	1	1	1	1	1	U	1	0	0	1	1	1	19
Hafer et al. [47]	0	1	1	1	1	1	U	1	0	0	1	1	1	19
Heiderscheit et al. [49]	0	1	1	1	1	1	U	1	1	U	1	1	1	19
Huang et al. [50]	0	1	1	1	1	1	U	1	1	U	1	1	1	19
Lenhart 2014 et al. [51]	0	1	1	1	1	1	U	1	1	U	1	1	1	19
Lenhart et al. [52]	0	1	1	1	1	1	U	1	1	U	1	1	1	19
Neal et al. [55]	0	1	1	1	1	1	U	1	0	0	1	1	1	18
Swinnen et al. [26]	0	1	1	1	1	1	U	1	1	U	1	1	1	19
Zimmerman et al. [57]	0	1	1	1	1	1	1	1	0	U	1	1	1	19
Adams et al. [43]	0	1	1	1	1	1	U	1	0	0	1	1	1	18
Bramah et al. [23]	0	1	1	1	1	1	U	1	0	0	1	1	1	18
Garofolini et al. [58]	0	1	1	1	1	1	U	1	0	0	1	1	1	18
Halvorsen et al. [48]	0	1	1	1	1	1	U	1	0	0	1	1	1	18
Hobara et al. [25]	0	1	1	1	1	1	U	1	1	U	1	1	1	18
Mercer et al. [53]	0	1	1	1	1	1	U	1	0	0	1	1	1	18
Morin et al. [54]	0	1	1	1	1	1	U	1	1	U	1	1	1	18
Quinn et al. [56]	0	1	1	1	1	1	U	1	0	0	1	1	1	18
Clarke et al. [45]	0	1	1	1	1	1	U	1	1	U	1	1	1	17
Connick and Li [46]	0	1	1	1	1	1	U	1	1	U	1	1	1	17
Dewolf and De Jaeger [24]	0	1	1	1	1	0	U	1	0	0	1	1	1	16

Item 5 assessed as yes = 2, partial = 1, no = 0, unclear = U. All other items are assessed as yes = 1, no = 0, unclear = U. High-quality scores ≥ 20, moderate -quality scores = 17 to 19, and low-quality scores ≤ 16

### Primary Outcomes

#### Injury

Two studies [23, 55] were identified evaluating pain and function with a change in running step rate over time periods of 4 weeks to 3 months. One study investigated the effects of a *10% increase in step rate* on *pain and function* in recreational runners with patellofemoral pain (1MQ [23]), providing limited evidence of improvements in total running distance per week, longest run pain-free, numeric pain rating scale, and Lower Extremity Functional Scale at 4 weeks and 3 months. The remaining study investigated the effects of a *7.5% increase in step rate* on *pain and function* in recreational runners with patellofemoral pain (1MQ [55]), providing limited evidence of improvements in average pain, worst pain, and the Kujala Scale at 6 weeks. No data pooling was possible for any injury variables. All SMDs and CI for the four variables and associated time periods are shown in Table 3.

**Table 3:** Single study results for injury variables

Variable	Post-intervention Time Frame	Step rate change	SMD ± 95% CI
Total running distance per week	4 weeks [23]	+ 10%	**1.26 [2.15, 0.37]**
	12 weeks [23]	+ 10%	**1.26 [2.15, 0.37]**
Longest run pain-free	4 weeks [23]	+ 10%	**2.05 [3.08, 1.03]**
	12 weeks [23]	+ 10%	**2.00 [3.01, 0.99]**
Numeric Rating Scale	4 weeks [23]	+ 10%	**2.68 [1.52, 3.83]**
	12 weeks [23]	+ 10%	**3.62 [2.24, 4.99]**
Lower Extremity Functional Scale	4 weeks [23]	+ 10%	**2.31 [3.39, 1.24]**
	12 weeks [23]	+ 10%	**3.42 [4.75, 2.09]**
Average Pain	6 weeks [55]	+ 7.5%	**1.55 [0.52, 2.58]**
Worst Pain	6 weeks [55]	+ 7.5%	**1.92 [0.82, 3.02]**
Kujala Scale	6 weeks [55]	+ 7.5%	− 0.68 [ − 1.59, 0.23]

SMD ± 95% CI in bold represent statistically significant results

#### Performance

Five studies [26, 42, 49, 50, 53] were identified evaluating the immediate differences in surrogate measures of performance with a change in running step rate.

##### Subjective Measures of Performance

Two studies were identified evaluating subjective measures of performance [49, 50]. In recreational runners, compared to running with a preferred step rate: very limited evidence indicated an increase in *rate of perceived exertion* (RPE) with a *10% increase in step rate*, but no differences were reported with a *5% increase in step rate*, or with *5% or 10% reductions in step rate* (1MQ [48]); and very limited evidence indicated an increase in self-reported *awkwardness* and *effort* with a *10% increase in step rate* (1MQ [49]).

##### Physiological Measures of Performance

Three studies were identified evaluating physiological measures of running performance [26, 42, 53]. In recreational runners, compared to running with a preferred step rate, very limited evidence indicated an increase in *VO*_*2*_* consumption* when running at 3.13 m/s and 3.58 m/s with a *15% decrease in step rate* [53]. Very limited evidence indicated no difference in *VO*_*2*_* consumption* when: running at 4.02 m/s with a *15% decrease in step rate* [53]; running at 3.13 m/s, 3.58 m/s and 4.02 m/s with a *15% increase in step rate* [53]; and, running at maximum speed for a 1-h run with a *4% and 8% increase or decrease in step rate* [42]. Very limited evidence indicated an increase in *metabolic energy consumption* with an *8% decrease*, *15% decrease and 15% increase in step rate*, while no difference was observed with an *8% increase in step rate* [26]. No data pooling was possible for any performance findings as no measure of performance was reported by multiple studies. All SMDs and CI from single studies are shown in Table 4.

**Table 4:** Single study results for performance variables

Variables		Preferred SR versus Increased SR		Preferred SR versus Reduced SR	
Performance	VO_2_ at 3.13 m/s	+ 15% [53]	− 0.21 ( − 1.09, 0.67)	− 15% [53]	** − 4.10 ( − 7.74, − 0.46)**
	VO_2_ at 3.58 m/s	+ 15% [53]	− 0.18 ( − 1.06, 0.70)	− 15% [53]	− 0.52 ( − 1.42, 0.37)
	VO_2_ at 4.02 m/s	+ 15% [53]	− 0.06 ( − 0.94, 0.81)	− 15% [53]	− 0.20 ( − 1.08, 0.68)
	VO_2_ at 60 min race pace	+ 4% (initial) [42]	− 0.23 ( − 0.92, 0.47)	− 4% (initial) [42]	− 0.26 ( − 0.95, 0.44)
		+ 4% (final) [42]	− 0.23 ( − 0.93, 0.46)	− 4% (final) [42]	− 0.23 ( − 0.92, 0.47)
		+ 8% (initial) [42]	− 0.45 ( − 1.15, 0.26)	− 8% (initial) [42]	− 0.45 ( − 1.16, 0.25)
		+ 8% (final) [42]	− 0.33 ( − 1.02, 0.37)	− 8% (final) [42]	− 0.34 ( − 1.04, 0.36)
	Rate of perceived exertion	+ 5% [49]	− 0.15 ( − 0.57, 0.26)	− 5% [49]	− 0.08 ( − 0.33, 0.50)
		+ 10% [49]	** − 0.49 ( − 0.91, − 0.07)**	− 10% [49]	0.00 ( − 0.41, 0.41)
	Awkwardness	+ 10% [50]	** − 0.72 ( − 1.38, − 0.06)**		
	Effort	+ 10% [50]	** − 0.69 ( − 1.34, − 0.03)**		
	Metabolic Energy Consumption	+ 8% [26]	− 0.38 [ − 1.08, 0.32]	− 8% [26]	− 0.70 [ − 1.41, 0.02]
		+ 15% [26]	** − 0.84 [ − 1.57, − 0.11]**	− 15% [26]	** − 1.61 [ − 2.42, − 0.80]**

SMD ± 95% CI are provided for each percentage increase or decrease in running step rate. SMD ± 95% CI presented in bold are statistically significant

### Secondary Outcome

**Fig. 2:**
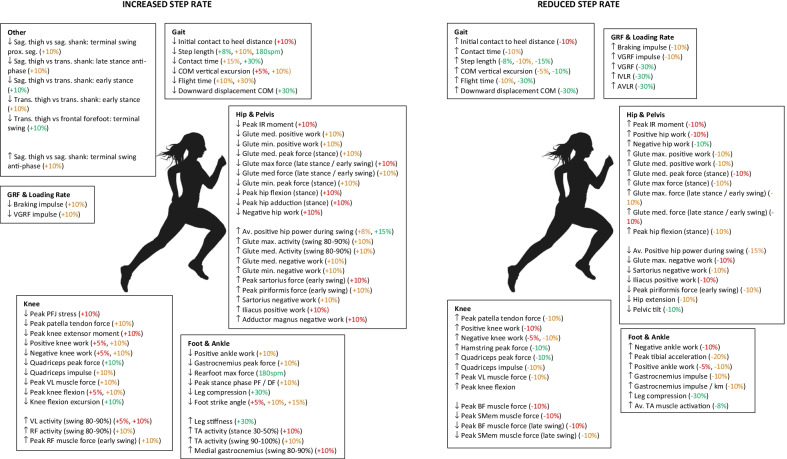
Significant biomechanical variables with changes in running step rate. Note: Changes in running step rate are provided in brackets next to each biomechanical variable (e.g. + 10% = 10% increase in habitual running step rate). Effect size of change is indicated by the colour of the text used to note the percentage change in running step rate (e.g. + 10% in red = small effect size with a 10% increase in habitual running step rate; orange = medium effect size; green = large effect size). *AV* average, *AVLR* average vertical loading rate, *BF* bicep femoris, *COM centre of mass, DF* dorsiflexion, *GLUTE MAX* gluteus maximus*, GLUTE MED* gluteus medius*, GLUTE MIN* gluteus minimus, *IR* internal rotation, *IVLR* instantaneous vertical loading rate, *PFJ* patellofemoral joint, *PF* plantarflexion, *PROX* proximal, *RF* rectus femoris, *SAG* sagittal, *SEG* segment, *SMEM* semimembranosus, *TA* tibialis anterior, *TRANS* transverse, *VGRF* verticl ground reaction orce, *VL* vasus lateralis

In the main manuscript, only the pooled results from two or more studies are presented for biomechanical variables. All SMDs and CI, including those from single studies are shown in Tables 5, 6, 7, 8, 9,10, 11, with all significant biomechanical findings additionally shown in Fig. 2. Unless stated otherwise, all reported findings are immediate effects to a change in running step rate.

**Table 5:** Pooled and single study results for spatiotemporal gait parameters

Variable		Preferred SR versus Increased SR		Preferred SR versus Reduced SR	
Spatiotemporal Gait Parameters	COM vertical excursion	+ 5% [49]	**0.53 (0.11, 0.95)**	− 5% [49]	** − 0.66 ( − 1.09, − 0.24)**
		+ 10% [49]	**1.15 (0.71, 1.60)**	− 10% [49]	** − 1.41 ( − 1.88, − 0.95)**
	Downward displacement of COM Contact time	+ 30% [54]	**1.92 (0.82, 3.01)**	− 30% [54]	** − 3.83 ( − 5.42, − 2.24**
		+ 5% [45]	0.41 ( − 0.48, 1.30)	− 5% [45]	− 0.71 ( − 1.62, 0.20)
		+ 8% [26]	0.50 ( − 0.20, 1.21)	− 8% [26]	− 0.28 ( − 0.98, 0.42)
		+ 10% [30, 45]	0.50 ( − 0.02, 1.03)	− 10% [30, 45]	** − 0.95 ( − 1.49, − 0.40)**
		+ 15% [26]	**0.92 (0.18, 1.65)**	− 15% [26]	− 0.35 ( − 1.05, 0.35)
		+ 30% [54]	**1.50 (0.48, 2.52)**	− 30% [54]	− 0.61 ( − 1.51, 0.30)
	Step length	+ 5% [49]	0.29 ( − 0.12, 0.71)	− 5% [49]	− 0.30 ( − 0.71, 0.12)
		+ 8% [26]	**1.72 (0.90, 2.55)**	− 8% [26]	** − 1.75 ( − 2.59, − 0.92)**
		+ 10% [30, 33, 44, 49]	**0.93 (0.49, 1.37)**	− 10% [24, 30, 44, 49]	** − 0.76 ( − 1.31, − 0.21)**
		180spm [57]	**4.69 (3.03, 6.35)**	− 15% [26]	** − 3.51 ( − 4.66, − 2.36)**
		+ 15% [26]	**2.73 (1.73, 3.72)**		
	COM to heel distance at IC	+ 5% [49]	0.36 ( − 0.06, 0.77)	− 5% [49]	− 0.26 ( − 0.67, 0.16)
		+ 10% [49]	**0.55 (0.13, 0.97)**	− 10% [49]	** − 0.53 ( − 0.95, − 0.11)**
	Flight time	+ 5% [45]	0.49 ( − 0.40, 1.38)	− 5% [45]	− 0.49 ( − 1.38, 0.40)
		+ 10% [45]	**0.98 (0.04, 1.92)**	− 10% [45]	** − 1.00 ( − 1.94, − 0.06)**
		+ 30% [54]	**1.13 (0.17, 2.09)**	− 30% [54]	** − 4.88 ( − 6.79, − 2.98)**
	Strike index	+ 10% [30]	− 0.53 ( − 1.18, 0.11)	− 10% [30]	− 0.12 ( − 0.75, 0.52)

SMD ± 95% CI are provided for each percentage increase or decrease in running step rate. SMD ± 95% CI presented in bold are statistically significant

Abbreviations: *COM* centre of mass, *IC* initial contact

**Table 6:** Pooled and single study results for ground reaction force and loading rate variables

Variable		Preferred SR versus Increased SR		Preferred SR versus Reduced SR	
Ground Reaction Force and Loading Rates	Average vertical loading rate	+ 7.5% (post 8 sessions) [40]	**1.25 (0.48, 2.01)**	− 15% [25]	− 0.46 ( − 1.35, 0.43)
		+ 7.5% (post 4 weeks) [40]	**1.37 (0.59, 2.14)**	− 30% [25]	** − 1.25 ( − 2.22, − 0.27)**
		+ 7.5% (post 12 weeks) [38]	0.81 ( − 0.03, 1.64)		
		+ 10% [41, 50]	0.24 ( − 0.23, 0.70)		
		+ 15% [25]	0.25 ( − 0.63, 1.13)		
		+ 30% [25]	0.24 ( − 0.64, 1.12)		
	Instantaneous vertical loading rate	+ 7.5% (post 8 sessions) [40]	**1.08 (0.33, 1.83)**	− 15% [25]	− 0.39 ( − 1.27, 0.50)
		+ 7.5% (post 4 weeks) [40]	**1.12 (0.37, 1.87)**	− 30% [25]	** − 1.23 ( − 2.20, − 0.25)**
		+ 7.5% (post 12 weeks) [38]	0.71 ( − 0.12, 1.54)		
		+ 10% [41, 49]	− 0.04 ( − 0.50, 0.42)		
		+ 15% [25]	0.34 ( − 0.55, 1.22)		
		+ 30% [25]	0.29 ( − 0.60, 1.17)		
	Vertical ground reaction force	+ 5% [49]	0.08 ( − 0.33, 0.50)	− 5% [49]	− 0.17 ( − 0.58, 0.25)
		+ 10% [33, 49]	0.24 ( − 0.11, 0.59)	− 10% [49]	− 0.24 ( − 0.65, 0.18)
		+ 30% [54]	0.29 ( − 0.59, 1.17)	− 30% [54]	** − 2.27 ( − 3.45, − 1.09)**
	Vertical ground reaction force impulse	+ 10% [30]	**1.15 (0.46, 1.84)**	− 10% [30]	** − 1.15 ( − 1.84, − 0.46)**
	Vertical impact peak	+ 7.5% (post 12 weeks) [38]	0.64 ( − 0.18, 1.47)	− 15% [25]	− 0.28 ( − 1.17, 0.60)
		+ 10%	0.07 ( − 0.57, 0.71)	− 30% [25]	− 0.90 ( − 1.83, 0.03)
		+ 15% [25]	0.15 ( − 0.73, 1.02)		
		+ 30% [25]	0.08 ( − 0.80, 0.96)		
	Time from initial contact to impact peak	+ 7.5% (post 12 weeks) [38]	− 0.21 ( − 1.01. 0.59)		
	Impact attenuation	+ 10% [44]	− 0.31 ( − 0.96, 0.33)	− 10% [44]	0.52 ( − 0.13, 1.17)
	Braking impulse	+ 5% [49]	0.38 ( − 0.04, 0.80)	− 5% [49]	− 0.35 ( − 0.76, 0.07)
		+ 10% [30, 49]	**0.64 (0.29, 1.00)**	− 10% [30, 49]	** − 0.73 ( − 1.08, − 0.37)**
	Head impact acceleration peak	+ 10% [31]	0.15 ( − 0.65, 0.95)	− 10% [31]	− 0.07 ( − 0.87, 0.73)
		+ 20% [31]	0.07 ( − 0.73, 0.87)	− 20% [31]	− 0.66 ( − 1.49, 0.16)
	Head active acceleration peak	+ 10% [31]	0.29 ( − 0.52, 1.09)	− 10% [31]	− 0.14 ( − 0.94, 0.66)
		+ 20% [31]	0.63 ( − 0.20, 1.45)	− 20% [31]	− 0.41 ( − 1.22, 0.40)
	Head signal power magnitude (3–8 Hz)	+ 10% [31]	0.26 ( − 0.55, 1.06)	− 10% [31]	− 0.18 ( − 0.98, 0.63)
		+ 20% [31]	0.53 ( − 0.29, 1.34)	− 20% [31]	− 0.36 ( − 1.17, 0.44)
	Head signal power magnitude (9–20 Hz)	+ 10% [31]	− 0.05 ( − 0.86, 0.75)	− 10% [31]	− 0.08 ( − 0.88, 0.72)
		+ 20% [31]	− 0.46 ( − 1.27, 0.36)	− 20% [31]	− 0.47 ( − 1.28, 0.34)
	Shock attenuation (active phase)	+ 10% [31]	− 0.60 ( − 1.42, 0.22)	− 10% [31]	0.15 ( − 0.65, 0.95)
		+ 20% [31]	− 0.82 ( − 1.66, 0.02)	− 20% [31]	− 0.01 ( − 0.81, 0.79)
	Shock attenuation (impact phase)	+ 10% [31]	− 0.16 ( − 0.96, 0.64)	− 10% [31]	0.28 ( − 0.52, 1.09)
		+ 20% [31]	− 0.44 ( − 1.25, 0.37)	− 20% [31]	0.15 ( − 0.66, 0.95)

SMD ± 95% CI are provided for each percentage increase or decrease in running step rate. SMD ± 95% CI presented in bold are statistically significant

**Table 7:** Pooled and single study results for kinetic, kinematic and muscle activation variables at the foot, ankle and lower leg

Variable		Preferred SR versus Increased SR		Preferred SR versus Reduced SR	
Kinetics	Peak tibial acceleration	+ 10% [31, 41, 44, 50]	0.06 ( − 0.29, 0.42)	− 10% [31, 44]	− 0.42 ( − 0.93, 0.08)
		+ 20% [31]	0.08 ( − 0.72, 0.88)	− 20% [31]	** − 1.13 ( − 2.01, − 0.26)**
	Tibial signal power magnitude (3–8 Hz)	+ 10% [31]	0.34 ( − 0.46, 1.15)	− 10% [31]	− 0.41 ( − 1.22, 0.40)
		+ 20% [31]	0.54 ( − 0.28, 1.36)	− 20% [31]	** − 0.92 ( − 1.76, − 0.07)**
	Tibial signal power magnitude (9–20 Hz)	+ 10% [31]	0.05 ( − 0.75, 0.85)	− 10% [31]	− 0.18 ( − 0.99, 0.62)
		+ 20% [31]	0.01 ( − 0.79, 0.81)	− 20% [31]	− 0.57 ( − 1.39, 0.25)
	Leg compression	+ 30% [54]	**3.83 (2.24, 5.42)**	− 30% [54]	** − 2.42 ( − 3.64, − 1.21)**
	Leg stiffness	+ 7.5% (at 12 − weeks) [38]	− 0.61 ( − 1.43, 0.22)		
		+ 30% [54]	** − 1.37 ( − 2.37, − 0.38)**	− 30% [54]	0.42 ( − 0.46, 1.31)
	Negative ankle work	+ 5% [49]	0.05 ( − 0.36, 0.46)	− 5% [49]	0.07 ( − 0.34, 0.49)
		+ 10% [44, 49]	0.01 ( − 0.33, 0.36)	− 10% [44, 49]	**0.38 (0.03, 0.73)**
	Positive ankle work	+ 5% [49]	0.33 ( − 0.09, 0.75)	− 5% [49]	** − 0.46 ( − 0.88, − 0.04)**
		+ 10% [49]	**0.74 (0.31, 1.16)**	− 10% [49]	** − 0.86 ( − 1.29, − 0.42)**
	Average positive ankle power (stance)	+ 8% [26]	0.18 [ − 0.52, 0.87]	− 8% [26]	− 0.39 [ − 1.09, 0.31]
		+ 15% [26]	0.54 [ − 0.17, 1.24]	− 15% [26]	** − 1.07 [ − 1.82, − 0.33]**
	Average positive ankle power (swing)	+ 8% [26]	0.00 [ − 0.69, 0.69]	− 8% [26]	**0.97 [0.24, 1.71]**
		+ 15% [26]	0.00 [ − 0.69, 0.69]	− 15% [26]	**0.97 [0.24, 1.71]**
	Plantarflexion moment	+ 10% [33]	− 0.06 ( − 0.70, 0.58)		
	Vertical foot velocity at initial contact	+ 5% [45]	0.12 ( − 0.75, 1.00)	− 5% [45]	− 0.37 ( − 1.26, 0.52)
		+ 10% [45]	0.14 ( − 0.74, 1.01)	− 10% [45]	− 0.54 ( − 1.43, 0.36)
	Horizontal foot velocity at initial contact	+ 5% [45]	− 0.15 ( − 1.03, 0.73)	− 5% [45]	0.14 ( − 0.74, 1.02)
		+ 10% [45]	− 0.11 ( − 0.98, 0.77)	− 10% [45]	0.17 ( − 0.70, 1.05)
	Average MGAS muscle activation	+ 8% [26]	0.11 [ − 0.58, 0.81]	− 8% [26]	0.31 [ − 0.38, 1.01]
		+ 15% [26]	0.00 [ − 0.69, 0.69]	− 15% [26]	0.13 [ − 0.56, 0.83]
	Average LGAS muscle activation	+ 8% [26]	0.00 [ − 0.69, 0.69]	− 8% [26]	− 0.10 [ − 0.79, 0.59]
		+ 15% [26]	0.05 [ − 0.65, 0.74]	− 15% [26]	− 0.28 [ − 0.98, 0.41]
	Average SOL muscle activation	+ 8% [26]	0.30 [ − 0.40, 1.00]	− 8% [26]	0.26 [ − 0.44, 0.95]
		+ 15% [26]	0.49 [ − 0.22, 1.19]	− 15% [26]	− 0.05 [ − 0.75, 0.64]
	Average TA muscle activation	+ 8% [26]	0.05 [ − 0.64, 0.74]	− 8% [26]	** − 2.78 [ − 3.78, − 1.77]**
		+ 15% [26]	− 0.05 [ − 0.74, 0.64]	− 15% [26]	− 0.05 [ − 0.74, 0.64]
	TA muscle activity – stance 0–15%	+ 5% [32]	− 0.33 ( − 0.74, 0.09)		
		+ 10% [32]	0.00 ( − 0.41, 0.41)		
	TA muscle activity—stance 30–50%	+ 5% [32]	− 0.20 ( − 0.61, 0.22)		
		+ 10% [32]	** − 0.59 ( − 1.02, − 0.17)**		
	TA muscle activity—swing 80–90%	+ 5% [32]	0.33 ( − 0.09, 0.75)		
		+ 10% [32]	0.33 ( − 0.09, 0.75)		
	TA muscle activity—swing 90–100%	+ 5% [32]	− 0.28 ( − 0.70, 0.13)		
		+ 10% [32]	** − 0.72 ( − 1.15, − 0.29)**		
	MGAS muscle activity—stance 0–15%	+ 5% [32]	0.00 ( − 0.41, 0.41)		
		+ 10% [32]	0.00 ( − 0.41, 0.41)		
	MGAS muscle activity—swing 80–90%	+ 5% [32]	− 0.22 ( − 0.63, 0.20)		
		+ 10% [32]	** − 0.45 ( − 0.86, − 0.03)**		
	MGAS muscle activity—swing 90–100%	+ 5% [32]	0.00 ( − 0.41, 0.41)		
		+ 10% [32]	− 0.40 ( − 0.82, 0.02)		
	GASTROC peak force	+ 10% [30]	**0.77 (0.11, 1.43)**	− 10% [30]	− 0.61 ( − 1.26, 0.04)
	GASTROC impulse	+ 10% [30]	0.33 ( − 0.31, 0.97)	− 10% [30]	** − 0.77 ( − 1.43, − 0.11)**
	GASTROC impulse/km	+ 10% [30]	0.60 ( − 0.05, 1.25)	− 10% [30]	** − 0.80 ( − 1.46, − 0.13)**
	Peak SOL muscle force	+ 10% [36]	0.37 ( − 0.14, 0.88)	− 10% [36]	− 0.51 ( − 1.03, 0.00)
	Peak MGAS muscle force—late stance / early swing (0–40%)	+ 10% [36]	0.14 ( − 0.37, 0.64)	− 10% [36]	0.29 ( − 0.22, 0.80)
	Peak MGAS muscle force—late swing (80–99%)	+ 10% [36]	− 0.28 ( − 0.79, 0.23)	− 10% [36]	0.26 ( − 0.25, 0.77)
	Peak TA muscle force	+ 10% [36]	0.11 ( − 0.40, 0.62)	− 10% [36]	0.33 ( − 0.18, 0.84)
	Rearfoot peak pressure	+ 5% [35, 39]	0.18 ( − 0.15, 0.51)	− 5% [35, 39]	− 0.14 ( − 0.48, 0.19)
		+ 10% [35]	0.31 ( − 0.19, 0.80)	− 10% [35]	− 0.35 ( − 0.84, 0.14)
		180spm [57]	0.30 ( − 0.50, 1.11)		
	Midfoot peak pressure	+ 5% [35]	0.08 ( − 0.41, 0.57)	− 5% [35]	0.03 ( − 0.46, 0.52)
		+ 10% [35]	0.25 ( − 0.24, 0.74)	− 10% [35]	0.05 ( − 0.44, 0.54)
		180spm [57]	0.14 ( − 0.66, 0.94)		
	Medial forefoot peak pressure	+ 5% [35]	− 0.06 ( − 0.55, 0.43)	− 5% [35]	0.03 ( − 0.46, 0.52)
		+ 10% [35]	− 0.05 ( − 0.54, 0.44)	− 10% [35]	− 0.02 ( − 0.51, 0.47)
	Lateral forefoot peak pressure	+ 5% [35]	0.06 ( − 0.43, 0.55)	− 5% [35]	0.03 ( − 0.46, 0.52)
		+ 10% [35]	0.07 ( − 0.42, 0.56)	− 10% [35]	0.09 ( − 0.40, 0.58)
	Hallux peak pressure	+ 5% [35]	0.04 ( − 0.45, 0.53)	− 5% [35]	− 0.09 ( − 0.58, 0.40)
		+ 10% [35]	0.06 ( − 0.43, 0.55)	− 10% [35]	− 0.08 ( − 0.57, 0.41)
	Rearfoot max force	+ 5% [35, 39]	0.17 ( − 0.16, 0.50)	− 5% [35, 39]	− 0.14 ( − 0.47, 0.19)
		+ 10% [35]	0.30 ( − 0.19, 0.79)	− 10% [35]	− 0.27 ( − 0.76, 0.22)
		180spm [57]	**1.29 (0.39, 2.18)**		
	Midfoot max force	+ 5% [35]	0.10 ( − 0.39, 0.59)	− 5% [35]	0.05 ( − 0.44, 0.54)
		+ 10% [35]	0.21 ( − 0.28, 0.70)	− 10% [35]	0.04 ( − 0.45, 0.53)
		180spm [57]	0.54 ( − 0.27, 1.36)		
	Medial forefoot max force	+ 5% [35]	0.00 ( − 0.49, 0.49)	− 5% [35]	− 0.01 ( − 0.50, 0.48)
		+ 10% [35]	0.00 ( − 0.49, 0.49)	− 10% [35]	− 0.01 ( − 0.50, 0.48)
	Lateral forefoot max force	+ 5% [35]	0.09 ( − 0.40, 0.58)	− 5% [35]	0.04 ( − 0.45, 0.53)
		+ 10% [35]	0.13 ( − 0.37, 0.62)	− 10% [35]	0.10 ( − 0.39, 0.59)
	Hallux max force	+ 5% [35]	0.05 ( − 0.44, 0.54)	− 5% [35]	− 0.11 ( − 0.60, 0.38)
		+ 10% [35]	0.11 ( − 0.38, 0.60)	− 10% [35]	− 0.13 ( − 0.62, 0.36)
	Rearfoot contact area	+ 5% [35]	0.06 ( − 0.43, 0.55)	− 5% [35]	0.00 ( − 0.49, 0.49)
		+ 10% [35]	0.12 ( − 0.37, 0.61)	− 10% [35]	− 0.04 ( − 0.53, 0.45)
	Midfoot contact area	+ 5% [35]	0.03 ( − 0.46, 0.52)	− 5% [35]	0.06 ( − 0.43, 0.55)
		+ 10% [35]	0.05 ( − 0.44, 0.54)	− 10% [35]	0.04 ( − 0.45, 0.53)
	Medial forefoot contact area	+ 5% [35]	0.00 ( − 0.49, 0.49)	− 5% [35]	0.04 ( − 0.45, 0.53)
		+ 10% [35]	0.00 ( − 0.49, 0.49)	− 10% [35]	0.04 ( − 0.45, 0.53)
	Lateral forefoot contact area	+ 5% [35]	0.02 ( − 0.47, 0.51)	− 5% [35]	0.04 ( − 0.45, 0.53)
		+ 10% [35]	0.02 ( − 0.47, 0.51)	− 10% [35]	0.04 ( − 0.45, 0.53)
	Hallux contact area	+ 5% [35]	0.00 ( − 0.49, 0.49)	− 5% [35]	0.00 ( − 0.49, 0.49)
		+ 10% [35]	0.00 ( − 0.49, 0.49)	− 10% [35]	0.05 ( − 0.44, 0.54)
	Rearfoot contact time	+ 5% [35, 39]	− 0.07 ( − 0.41, 0.26)	− 5% [35, 39]	− 0.23 ( − 0.56, 0.10)
		+ 10% [35]	− 0.16 ( − 0.65, 0.33)	− 10% [35]	− 0.35 ( − 0.85, 0.14)
	Midfoot contact time	+ 5% [35]	− 0.28 ( − 0.77, 0.22)	− 5% [35]	− 0.27 ( − 0.76, 0.23)
		+ 10% [35]	− 0.15 ( − 0.64, 0.34)	− 10% [35]	− 0.26 ( − 0.75, 0.23)
	Medial forefoot contact time	+ 5% [35]	− 0.30 ( − 0.79, 0.19)	− 5% [35]	− 0.08 ( − 0.57, 0.41)
		+ 10% [35]	− 0.26 ( − 0.75, 0.23)	− 10% [35]	− 0.13 ( − 0.63, 0.36)
	Lateral forefoot contact time	+ 5% [35]	− 0.27 ( − 0.76, 0.22)	− 5% [35]	− 0.10 ( − 0.59, 0.39)
		+ 10% [35]	− 0.24 ( − 0.73, 0.26)	− 10% [35]	− 0.15 ( − 0.64, 0.34)
	Hallux contact time	+ 5% [35]	− 0.19 ( − 0.68, 0.30)	− 5% [35]	− 0.04 ( − 0.53, 0.45)
		+ 10% [35]	− 0.13 ( − 0.62, 0.36)	− 10% [35]	− 0.08 ( − 0.57, 0.41)
	Total foot contact time	+ 5% [39]	0.30 ( − 0.15, 0.75)	− 5% [39]	− 0.20 ( − 0.65, 0.25)
	Medial forefoot contact time	+ 5% [39]	0.21 ( − 0.24, 0.66)	− 5% [39]	− 0.21 ( − 0.66, 0.24)
	Central forefoot contact time	+ 5% [39]	0.19 ( − 0.26, 0.64)	− 5% [39]	− 0.25 ( − 0.70, 0.20)
	Lateral forefoot contact time	+ 5% [39]	0.26 ( − 0.19, 0.72)	− 5% [39]	− 0.15 ( − 0.60, 0.30)
	Heel force time integral	+ 5% [39]	0.26 ( − 0.19, 0.71)	− 5% [39]	− 0.04 ( − 0.49, 0.41)
	Medial forefoot force time integral	+ 5% [39]	0.17 ( − 0.28, 0.62)	− 5% [39]	− 0.16 ( − 0.61, 0.29)
	Central forefoot force time integral	+ 5% [39]	0.21 ( − 0.24, 0.66)	− 5% [39]	− 0.21 ( − 0.66, 0.24)
	Lateral forefoot force time integral	+ 5% [39]	0.10 ( − 0.35, 0.55)	− 5% [39]	− 0.10 ( − 0.55, 0.35)
	Total foot peak force	+ 5% [39]	0.12 ( − 0.33, 0.57)	− 5% [39]	0.05 ( − 0.40, 0.50)
	Medial forefoot peak force	+ 5% [39]	0.06 ( − 0.39, 0.51)	− 5% [39]	− 0.11 ( − 0.56, 0.34)
	Central forefoot peak force	+ 5% [39]	0.05 ( − 0.40, 0.50)	− 5% [39]	− 0.05 ( − 0.50, 0.40)
	Lateral forefoot peak force	+ 5% [39]	0.00 ( − 0.45, 0.45)	− 5% [39]	− 0.07 ( − 0.52, 0.38)
	Total foot peak pressure	+ 5% [39]	0.08 ( − 0.37, 0.53)	− 5% [39]	− 0.20 ( − 0.65, 0.25)
	Medial forefoot peak pressure	+ 5% [39]	0.18 ( − 0.27, 0.63)	− 5% [39]	0.08 ( − 0.37, 0.53)
	Central forefoot peak pressure	+ 5% [39]	0.08 ( − 0.37, 0.53)	− 5% [39]	− 0.06 ( − 0.51, 0.39)
	Lateral forefoot peak pressure	+ 5% [39]	0.29 ( − 0.16, 0.74)	− 5% [39]	0.20 ( − 0.25, 0.66)
	Heel pressure time integral	+ 5% [39]	0.28 ( − 0.17, 0.73)	− 5% [39]	− 0.06 ( − 0.51, 0.39)
	Medial forefoot pressure time integral	+ 5% [39]	0.28 ( − 0.17, 0.73)	− 5% [39]	0.02 ( − 0.43, 0.47)
	Central forefoot pressure time integral	+ 5% [39]	0.18 ( − 0.27, 0.63)	− 5% [39]	− 0.15 ( − 0.60, 0.30)
	Lateral forefoot pressure time integral	+ 5% [39]	0.33 ( − 0.12, 0.78)	− 5% [39]	0.03 ( − 0.42, 0.48)
	Forefoot max force	180spm [57]	0.78 ( − 0.05, 1.62)		
	Forefoot max pressure	180spm [57]	0.47 ( − 0.34, 1.28)		
Kinematics	Average ankle PF/DF at IC	+ 5% [45]	0.05 ( − 0.83, 0.92)	− 5% [45]	0.13 ( − 0.75, 1.01)
		+ 10% [34, 45]	0.23 ( − 0.20, 0.57)	− 10% [45]	− 0.26 ( − 1.14, 0.63)
	Foot strike angle	+ 5% [22, 49]	**0.39 (0.09, 0.69)**	− 5% [49]	− 0.13 ( − 0.55, 0.28)
		+ 7.5% (at 12 − weeks) [38]	**0.99 (0.13, 1.84)**		
		+ 10% [22, 33, 49]	**0.62 (0.34, 0.90)**	− 10% [49]	− 0.27 ( − 0.68, 0.15)
		+ 15% [22]	**1.19 (0.71, 1.67)**		
	Average ankle PF/DF during stance	+ 10% [34]	0.06 ( − 0.43, 0.56)		
	Peak ankle PF/DF during stance	+ 10% [34]	**0.85 (0.33, 1.37)**		
	Max DF during stance	+ 7.5% (at 12 − weeks) [38]	− 0.09 ( − 0.89, 0.71)		
	Peak rearfoot eversion angle	+ 10% [47]	0.04 ( − 0.84, 0.92)		
	Peak rearfoot eversion % of GC	+ 10% [47]	0.08 ( − 0.80, 0.96)		
	Peak shank IR angle	+ 10% [47]	0.02 ( − 0.86, 0.89)		
	Peak shank IR % of GC	+ 10% [47]	− 0.87 ( − 1.8, 0.05)		

SMD ± 95% CI are provided for each percentage increase or decrease in running step rate. SMD ± 95% CI presented in bold are statistically significant

Abbreviations: *DF* dorsiflexion, *GASTROC* gastrocnemius, *GC* gait cycle, *IC* initial contact, *LGAS* lateral gastrocnemius, *MGAS* medial gastrocnemius, *PF* plantarflexion, *SOL* soleus, *TA* tibialis anterior

**Table 8:** Pooled and single study results for kinetic, kinematic and muscle activation variables at the knee

Variable		Preferred SR versus Increased SR		Preferred SR versus Reduced SR	
Kinetics	Peak patellar tendon force	+ 10% [36]	**0.68 (0.16, 1.21)**	− 10% [36]	** − 0.72 ( − 1.24, − 0.19)**
	Peak PFJ stress	+ 10% [29, 33]	**0.56 (0.07, 1.05)**		
	Peak PFJ reaction force	+ 10% [29]	0.66 ( − 0.07, 1.40)		
	PFJS-time integral	+ 10% [33]	0.65 ( − 0.01, 1.30)		
	PFJS-time integral/km	+ 10% [5]	0.49 ( − 0.16, 1.14)		
	Peak knee extensor moment	+ 5% [49]	0.17 ( − 0.25, 0.58)	− 5% [49]	− 0.33 ( − 0.75, 0.09)
		+ 10% [29, 33, 49]	**0.50 (0.18, 0.81)**	− 10% [49]	− 0.33 ( − 0.75, 0.09)
	Negative knee work	+ 5% [49]	** − 0.51 ( − 0.93, − 0.09)**	− 5% [49]	**0.50 (0.08, 0.91)**
		+ 10% [44, 49]	** − 0.84 ( − 1.20, − 0.48)**	− 10% [44, 49]	**0.88 (0.52, 1.25)**
	Positive knee work	+ 5% [49]	**0.49 (0.07, 0.91)**	− 5% [49]	− 0.21 ( − 0.62, 0.21)
		+ 10% [49]	**0.75 (0.32, 1.18)**	− 10% [49]	− **0.53 ( − 0.95, − 0.10)**
	Average positive knee power (stance)	+ 8% [26]	0.41 [ − 0.29, 1.11]	− 8% [26]	− 0.22 [ − 0.92, 0.47]
		+ 15% [26]	0.68 [ − 0.04, 1.39]	− 15% [26]	− 0.47 [ − 1.17, 0.24]
	Average positive knee power (swing)	+ 8% [26]	0.22 [ − 0.48, 0.91]	− 8% [26]	0.00 [ − 0.69, 0.69]
		+ 15% [26]	0.22 [ − 0.48, 0.91]	− 15% [26]	0.22 [ − 0.48, 0.91]
	Eccentric knee work per stance	+ 7.5% (8 sessions) [40]	**1.02 (0.27, 1.76)**		
		+ 7.5% (4 − weeks) [40]	**0.79 (0.07, 1.51)**		
	Eccentric knee work per km	+ 7.5% (8 sessions) [40]	0.02 ( − 0.68, 0.71)		
		+ 7.5% (4 − weeks) [40]	0.57 ( − 0.14, 1.28)		
	Max knee flexion velocity during stance	+ 5% [45]	0.28 ( − 0.61, 1.16)	− 5% [45]	− 0.28 ( − 1.17, 0.60)
		+ 10% [45]	0.67 ( − 0.24, 1.57)	− 10% [45]	− 0.92 ( − 1.86, 0.01)
	VL muscle activity—stance 0–15%	+ 5% [32]	0.18 ( − 0.24, 0.59)		
		+ 10% [32]	0.17 ( − 0.25, 0.58)		
	VL muscle activity—swing 80–90%	+ 5% [32]	** − 0.44 ( − 0.86, − 0.02)**		
		+ 10% [32]	** − 0.43 ( − 0.85, − 0.01)**		
	VL muscle activity—swing 90–100%	+ 5% [32]	0.12 ( − 0.30, 0.53)		
		+ 10% [32]	0.11 ( − 0.30, 0.52)		
	RF muscle activity—stance 0–15%	+ 5% [32]	0.09 ( − 0.32, 0.51)		
		+ 10% [32]	0.00 ( − 0.41, 0.41)		
	RF muscle activity—stance 30–50%%	+ 5% [32]	0.00 ( − 0.41, 0.41)		
		+ 10% [32]	− 0.42 ( − 0.84, 0.00)		
	RF muscle activity—swing 80–90%	+ 5% [32]	0.00 ( − 0.41, 0.41)		
		+ 10% [32]	− 0.23 ( − 0.64, 0.18)		
	RF muscle activity—swing 90–100%	+ 5% [32]	− 0.39 ( − 0.81, 0.03)		
		+ 10% [32]	** − 0.78 ( − 1.21, − 0.35)**		
	LHAMS muscle activity—stance 0–15%	+ 5% [32]	0.00 ( − 0.41, 0.41)		
		+ 10% [32]	− 0.15 ( − 0.56, 0.27)		
	LHAMS muscle activity—swing 70–80%	+ 5% [32]	0.12 ( − 0.29, 0.54)		
		+ 10% [32]	− 0.26, − 0.68, 0.15)		
	LHAMS muscle activity—swing 80–90%	+ 5% [32]	− 0.10 ( − 0.51, 0.31)		
		+ 10% [32]	− 0.16 ( − 0.58, 0.25)		
	LHAMS muscle activity—swing 90–100%	+ 5% [32]	− 0.10 ( − 0.52, 0.31)		
		+ 10% [32]	0.00 ( − 0.41, 0.41)		
	MHAMS muscle activity—stance 0–15%	+ 5% [32]	− 0.22 ( − 0.63, 0.20)		
		+ 10% [32]	− 0.20 ( − 0.61, 0.22)		
	MHAMS muscle activity—swing 70–80%	+ 5% [32]	− 0.12 ( − 0.54, 0.29)		
		+ 10% [32]	− 0.40 ( − 0.81, 0.02)		
	MHAMS muscle activity—swing 80–90%	+ 5% [32]	0.09 ( − 0.33, 0.50)		
		+ 10% [32]	− 0.08 ( − 0.49, 0.34)		
	MHAMS muscle activity—swing 90–100%	+ 5% [32]	− 0.20 ( − 0.61, 0.22)		
		+ 10% [32]	− 0.10 ( − 0.52, 0.31)		
	BF positive work	+ 10% [51]	0.00 ( − 0.51, 0.51)	− 10% [51]	0.06 ( − 0.44, 0.57)
	BF negative work	+ 10% [51]	0.25 ( − 0.26, 0.75)	− 10% [51]	− 0.07 ( − 0.57, 0.44)
	SMEM positive work	+ 10% [51]	0.00 ( − 0.51, 0.51)	− 10% [51]	− 0.27 ( − 0.78, 0.23)
	SMEM negative work	+ 10% [51]	0.44 ( − 0.07, 0.95	− 10% [51]	− 0.24 ( − 0.75, 0.27)
	RF positive work	+ 10% [51]	0.29 ( − 0.22, 0.80)	− 10% [51]	− 0.22 ( − 0.73, 0.29)
	RF negative work	+ 10% [51]	− 0.12 ( − 0.62, 0.39)	− 10% [51]	0.22 ( − 0.29, 0.73)
	Hamstring peak force	+ 10% [30]	0.06 ( − 0.57, 0.70)	− 10% [30]	** − 1.28 ( − 1.98, − 0.57)**
	Hamstring impulse	+ 10% [30]	0.28 ( − 0.36, 0.92)	− 10% [30]	− 0.24 ( − 0.88, 0.39)
	Hamstring impulse/km	+ 10% [30]	0.00 ( − 0.64, 0.64)	− 10% [30]	− 0.11 ( − 0.74, 0.53)
	Quadriceps peak force	+ 10% [30]	**1.87 (1.09, 2.64)**	− 10% [30]	** − 1.82 ( − 2.59, − 1.05)**
	Quadriceps impulse	+ 10% [30]	**0.71 (0.05, 1.37)**	− 10% [30]	** − 0.89 ( − 1.56, − 0.22)**
	Quadriceps impulse/km	+ 10% [30]	0.31 ( − 0.33, 0.95)	− 10% [30]	− 0.46 ( − 1.10, 0.19)
	Peak VL muscle force	+ 10% [36]	**0.76 (0.24, 1.29)**	− 10% [36]	** − 0.81 ( − 1.34, − 0.28)**
	Peak RF muscle force	+ 10% [36]	− 0.21 ( − 0.72, 0.30)	− 10% [36]	0.44 ( − 0.08, 0.95)
	Peak BF muscle force	+ 10% [36]	0.00 ( − 0.51, 0.51)	− 10% [36]	**0.56 (0.05, 1.08)**
	Peak SMEM muscle force	+ 10% [36]	− 0.20 ( − 0.71, 0.31)	− 10% [36]	**0.59 (0.07, 1.11)**
	Peak BF muscle force—stance	+ 10% [50]	− 0.24 ( − 0.75, 0.27)	− 10% [50]	0.18 ( − 0.33, 0.68)
	Peak BF muscle force—late swing	+ 10% [50]	− 0.06 ( − 0.57, 0.44)	− 10% [50]	**0.53 (0.02, 1.05)**
	Peak SMEM muscle force—stance	+ 10% [50]	− 0.27 ( − 0.78, 0.24)	− 10% [50]	0.20 ( − 0.31, 0.71)
	Peak SMEM muscle force—late swing	+ 10% [51]	− 0.19 ( − 0.70, 0.32)	− 10% [51]	**0.60 (0.08, 1.12)**
	Peak RF muscle force—stance	+ 10% [51]	0.37 ( − 0.14, 0.88)	− 10% [51]	− 0.36 ( − 0.87, 0.15)
	Peak RF muscle force—early swing	+ 10% [51]	** − 0.68 ( − 1.20, − 0.15)**	− 10% [51]	0.47 ( − 0.04, 0.98)
Kinematics	Peak knee flexion angle	+ 5% [49]	**0.47 (0.05, 0.89)**	− 5% [49]	− 0.37 ( − 0.78, 0.05)
		+ 7.5% (at 12 weeks) [38]	0.39 ( − 0.42, 1.20)		
		+ 10% [29, 33, 34, 47, 49]	**0.66 (0.40, 0.92)**	− 10% [49]	** − 0.92 ( − 1.35, − 0.48)**
		+ 10% (at 4 − weeks) [23]	**0.91 (0.06, 1.76)**		
		+ 10% (at 12 − weeks) [23]	0.60 ( − 0.22, 1.43)		
	Peak knee flexion % of GC	+ 10% [47]	− 0.44 ( − 1.33, 0.45)		
	Knee flexion excursion	+ 10% [47]	**1.67 (0.62, 2.73)**		
	Average knee ADD/ABD at IC	+ 10% [34]	− 0.10 ( − 0.60, 0.40)		
	Average knee ADD/ABD during stance phase	+ 10% [34]	− 0.06 ( − 0.56, 0.44)		
	Peak knee ADD/ABD during stance phase	+ 10% [34]	0.17 ( − 0.33, 0.66)		
	Average knee ER at IC	+ 10% [34]	− 0.11 ( − 0.61, 0.39)		
	Average knee ER during stance phase	+ 10% [34]	− 0.03 ( − 0.53, 0.46)		
	Peak knee ER during stance phase	+ 10% [34]	− 0.09 ( − 0.59, 0.41)		
	Peak knee IR angle	+ 10% [46]	− 0.14 ( − 1.02, 0.73)		
	Peak knee IR % of GC	+ 10% [47]	− 0.29 ( − 1.17, 0.59)		
	Average knee flexion at IC	+ 5% [45, 49]	− 0.19 ( − 0.57, 0.18)	− 5% [45, 49]	0.15 ( − 0.22, 0.53)
		+ 10% [34, 45, 49]	− 0.23 ( − 0.53, 0.07)	− 10% [45, 49]	0.18 ( − 0.20, 0.55)
	Average knee flexion during stance phase	+ 10% [34]	0.28 ( − 0.22, 0.78)		

SMD ± 95% CI are provided for each percentage increase or decrease in running step rate. SMD ± 95% CI presented in bold are statistically significant

Abbreviations: *ABD* abduction, *ADD* adduction, *BF* bicep femoris, *ER* external rotation, *GC* gait cycle, *IC* initial contact, *LHAMS* lateral hamstring, *MHAMS* medial hamstring, *PFJ* patellofemoral joint, *PFJS* patellofemoral joint stress, *RF* rectus femoris, *SMEM* semimembranosus, *VL* vastus lateralis

**Table 9:** Pooled and single study results for kinetic, kinematic and muscle activation variables at the hip

Variable		Preferred SR versus Increased SR		Preferred SR versus Reduced SR	
Kinetics	Hip extension moment at IC	+ 5% [49]	− 0.20 ( − 0.61, 0.22)	− 5% [49]	0.00 ( − 0.41, 0.41)
		+ 10% [49]	− 0.20 ( − 0.61, 0.22)	− 10% [49]	0.20 ( − 0.22, 0.61)
	Hip extensor moment during stance phase	+ 10% [33]	0.18 ( − 0.45, 0.82)		
	Peak hip abduction moment	+ 5% [49]	0.00 ( − 0.41, 0.41)	− 5% [49]	0.00 ( − 0.41, 0.41)
		+ 10% [49]	0.25 ( − 0.17, 0.66)	− 10% [49]	− 0.22 ( − 0.63, 0.20)
	Peak hip IR moment	+ 5% [49]	0.00 ( − 0.41, 0.41)	− 5% [49]	0.00 ( − 0.41, 0.41)
		+ 10% [49]	**0.50 (0.08, 0.92)**	− 10% [49]	** − 0.50 ( − 0.92, − 0.08)**
	Negative hip work	+ 5% [49]	− 0.25 ( − 0.66, 0.17)	− 5% [49]	0.26 ( − 0.15, 0.68)
		+ 10% [44, 49]	** − 0.55 ( − 0.91, − 0.20)**	− 10% [44, 49]	** − 0.67 ( − 1.02, − 0.31)**
	Positive hip work	+ 5% [49]	0.04 ( − 0.37, 0.46)	− 5% [49]	− 0.20 ( − 0.61, 0.22)
		+ 10% [49]	0.13 ( − 0.28, 0.54)	− 10% [49]	** − 0.49 ( − 0.91, − 0.07)**
	Average positive hip power (stance)	+ 8% [26]	0.14 [ − 0.55, 0.83]	− 8% [26]	− 0.07 [ − 0.76, 0.62]
		+ 15% [26]	0.31 [ − 0.39, 1.01]	− 15% [26]	− 0.42 [ − 1.12, 0.28]
	Average positive hip power (swing)	+ 8% [26]	** − 0.82 [ − 1.55, − 0.10]**	− 8% [26]	0.63 [ − 0.08, 1.35]
		+ 15% [26]	** − 1.61 [ − 2.42, − 0.80]**	− 15% [26]	**1.13 [0.38, 1.88]**
	GMAX muscle activity—stance 0–15%	+ 5% [32]	− 0.11 ( − 0.52, 0.30)		
		+ 10% [32]	− 0.33 ( − 0.74, 0.09)		
	GMAX muscle activity—swing 80–90%	+ 5% [32]	− 0.15 ( − 0.57, 0.26)		
		+ 10% [32]	** − 0.60 ( − 1.02, − 0.18)**		
	GMAX muscle activity—swing 90–100%	+ 5% [32]	− 0.17 ( − 0.58, 0.24)		
		+ 10% [32]	− 0.34 ( − 0.76, 0.07)		
	GMED muscle activity—stance 0–15%	+ 5% [32]	− 0.10 ( − 0.51, 0.31)		
		+ 10% [32]	− 0.30 ( − 0.71, 0.12)		
	GMED muscle activity—swing 80–90%	+ 5% [32]	− 0.33 ( − 0.75, 0.09)		
		+ 10% [32]	** − 0.70 ( − 1.13, − 0.27)**		
	GMED muscle activity—swing 90–100%	+ 5% [32]	− 0.12 ( − 0.54, 0.29)		
		+ 10% [32]	** − 0.46 ( − 0.88, − 0.04)**		
	GMAX positive work	+ 10% [51]	0.37 ( − 0.14, 0.88)	− 10% [51]	** − 0.64 ( − 1.16, − 0.12)**
	GMAX negative work	+ 10% [51]	− 0.37 ( − 0.88, 0.14)	− 10% [51]	**0.54 (0.02, 1.05)**
	GMED positive work	+ 10% [51]	**0.88 (0.34, 1.41)**	− 10% [51]	** − 0.88 ( − 1.41, − 0.35)**
	GMED negative work	+ 10% [51]	** − 0.85 ( − 1.37, − 0.32)**	− 10% [51]	0.28 ( − 0.23, 0.79)
	GMIN positive work	+ 10% [51]	**0.72 (0.20, 1.24)**	− 10% [51]	− 0.42 ( − 0.94, 0.09)
	GMIN negative work	+ 10% [51]	** − 0.69 ( − 1.21, − 0.17)**	− 10% [51]	0.00 ( − 0.51, 0.51)
	TFL positive work	+ 10% [51]	0.20 ( − 0.31, 0.70)	− 10% [51]	0.00 ( − 0.51, 0.51)
	TFL negative work	+ 10% [51]	− 0.28 ( − 0.79, 0.23)	− 10% [51]	0.00 ( − 0.51, 0.51)
	SART positive work	+ 10% [51]	− 0.33 ( − 0.84, 0.18)	− 10% [51]	0.00 ( − 0.51, 0.51)
	SART negative work	+ 10% [51]	** − 0.62 ( − 1.14, − 0.11)**	− 10% [51]	**0.62 (0.11, 1.14)**
	Psoas positive work	+ 10% [51]	0.00 ( − 0.51, 0.51)	− 10% [51]	0.04 ( − 0.47, 0.54)
	Psoas negative work	+ 10% [51]	− 0.25 ( − 0.76, 0.25)	− 10% [51]	0.23 ( − 0.28, 0.74)
	Iliacus positive work	+ 10% [51]	** − 0.55 ( − 1.06, − 0.03)**	− 10% [51]	**0.58 (0.06, 1.10)**
	Iliacus negative work	+ 10% [51]	− 0.13 ( − 0.64, 0.38)	− 10% [51]	0.33 ( − 0.18, 0.84)
	ADDMAG positive work	+ 10% [51]	0.21 ( − 0.30, 0.72)	− 10% [51]	− 0.21 ( − 0.72, 0.30)
	ADDMAG negative work	+ 10% [51]	** − 0.56 ( − 1.07, − 0.04)**	− 10% [51]	0.22 ( − 0.29, 0.73)
	ADDBREV positive work	+ 10% [51]	0.07 ( − 0.43, 0.58)	− 10% [51]	0.21 ( − 0.30, 0.72)
	ADDBREV negative work	+ 10% [51]	− 0.49 ( − 1.00, 0.02)	− 10% [51]	0.20 ( − 0.31, 0.70)
	ADDLONG positive work	+ 10% [51]	0.10 ( − 0.40, 0.61)	− 10% [51]	0.21 ( − 0.30, 0.72)
	ADDLONG negative work	+ 10% [51]	− 0.44 ( − 0.95, 0.08)	− 10% [51]	0.36 ( − 0.15, 0.87)
	Piriformis positive work	+ 10% [51]	0.28 ( − 0.23, 0.79)	− 10% [51]	− 0.44 ( − 0.95, 0.08)
	Piriformis negative work	+ 10% [51]	− 0.22 ( − 0.73, 0.29)	− 10% [51]	0.36 ( − 0.15, 0.87)
	Peak GMED muscle force—late stance / early swing (0–40%)	+ 10% [36]	**0.90 (0.37, 1.43)**	− 10% [36]	** − 0.54 ( − 1.05, − 0.02)**
	Peak GMED muscle force—late swing (80–99%)	+ 10% [36]	− 0.32 ( − 0.83, 0.19)	− 10% [36]	0.49 ( − 0.02, 1.01)
	Peak GMAX muscle force—late stance / early swing (0–40%)	+ 10% [36]	**0.57 (0.05, 1.09)**	− 10% [36]	** − 0.78 ( − 1.30, − 0.25)**
	Peak GMAX muscle force—late swing (80–99%)	+ 10% [36]	− 0.07 ( − 0.58, 0.43)	− 10% [36]	0.33 ( − 0.18, 0.84)
	Peak GMED muscle force—stance	+ 10% [51]	**0.88 (0.34, 1.41)**	− 10% [51]	** − 0.54 ( − 1.05, − 0.02)**
	Peak GMED muscle force—late swing	+ 10% [51]	− 0.31 ( − 0.82, 0.20)	− 10% [51]	0.44 ( − 0.07, 0.96)
	Peak GMIN muscle force—stance	+ 10% [51]	**0.61 (0.09, 1.13)**	− 10% [51]	− 0.32 ( − 0.83, 0.19)
	Peak GMIN muscle force—early swing	+ 10% [51]	0.08 ( − 0.43, 0.59)	− 10% [51]	0.41 ( − 0.10, 0.92)
	Peak GMIN muscle force—late swing	+ 10% [51]	− 0.28 ( − 0.79, 0.23)	− 10% [51]	0.40 ( − 0.11, 0.91)
	Peak GMAX muscle force—stance	+ 10% [51]	0.42 ( − 0.09, 0.93)	− 10% [51]	** − 0.71 ( − 1.24, − 0.19)**
	Peak GMAX muscle force—late swing	+ 10% [51]	− 0.07 ( − 0.58, 0.43)	− 10% [51]	0.33 ( − 0.18, 0.84)
	Peak TFL muscle force—early swing	+ 10% [51]	− 0.36 ( − 0.87, 0.15)	− 10% [51]	0.37 ( − 0.14, 0.88)
	Peak SART muscle force—early swing	+ 10% [51]	** − 0.54 ( − 1.06, − 0.03)**	− 10% [51]	0.35 ( − 0.16, 0.86)
	Peak psoas muscle force—early swing	+ 10% [51]	− 0.05 ( − 0.56, 0.45)	− 10% [51]	0.12 ( − 0.38, 0.63)
	Peak iliacus muscle force—early swing	+ 10% [51]	− 0.29 ( − 0.80, 0.22)	− 10% [51]	0.44 ( − 0.08, 0.95)
	Peak ADDMAG muscle force—stance	+ 10% [51]	0.08 ( − 0.42, 0.59)	− 10% [51]	− 0.34 ( − 0.85, 0.17)
	Peak ADDBREV muscle force—early swing	+ 10% [51]	− 0.18 ( − 0.69, 0.32)	− 10% [51]	0.32 ( − 0.19, 0.83)
	Peak ADDLONG muscle force—early swing	+ 10% [51]	− 0.11 ( − 0.61, 0.40)	− 10% [51]	0.05 ( − 0.45, 0.56)
	Peak piriformis muscle force—stance	+ 10% [51]	0.45 ( − 0.06, 0.96)	− 10% [51]	− 0.47 ( − 0.98, 0.05)
	Peak piriformis muscle force—early swing	+ 10% [51]	** − 0.65 ( − 1.17, − 0.13)**	− 10% [51]	**0.63 (0.11, 1.15)**
	Peak piriformis muscle force—late swing	+ 10% [51]	− 0.09 ( − 0.60, 0.41)	− 10% [51]	0.15 ( − 0.36, 0.66)
Kinematics	Average hip flexion at IC	+ 5% [45]	0.06 ( − 0.81, 0.94)	− 5% [45]	− 0.03 ( − 0.91, 0.85)
		+ 10% [34, 45]	0.14 ( − 0.29, 0.57)	− 10% [45]	− 0.35 ( − 1.23, 0.54)
	Average hip flexion during stance phase	+ 10% [34]	0.07 ( − 0.42, 0.57)		
	Peak hip flexion during stance phase	+ 5% [49]	0.25 ( − 0.16, 0.67)	− 5% [49]	− 0.21 ( − 0.62, 0.20)
		+ 7.5% (post 12 weeks) [38]	0.05 ( − 0.75, 0.85)		
		+ 10% [34, 49]	**0.42 (0.10, 0.75)**	− 10% [49]	** − 0.71 ( − 1.13, − 0.28)**
	Average hip adduction at IC	+ 10% [34]	− 0.03 ( − 0.53, 0.47)		
	Average hip adduction during stance phase	+ 10% [34]	0.00 ( − 0.49, 0.50)		
	Peak hip adduction during stance phase	+ 5% [49]	0.28 ( − 0.14, 0.69)	− 5% [49]	− 0.12 ( − 0.53, 0.29)
		+ 7.5% (post 8 sessions) [40]	0.72 ( − 0.00, 1.44)		
		+ 7.5% (post 4 weeks) [40]	0.59 ( − 0.12, 1.30)		
		+ 10% [34, 41, 49]	**0.40 (0.11, 0.69)**	− 10% [49]	− 0.26 ( − 0.67, 0.16)
	Average hip IR at IC	+ 10% [34]	0.18 ( − 0.32, 0.68)		
	Average hip IR during stance phase	+ 10% [34]	0.02 ( − 0.48, 0.52)		
	Peak hip IR during stance phase	+ 5% [49]	0.02 ( − 0.39, 0.44)	− 5% [49]	− 0.09 ( − 0.50, 0.32)
		+ 10% [34, 49]	0.07 ( − 0.25, 0.38)	− 10% [49]	− 0.19 ( − 0.61, 0.22)
		+ 10% (post 4 weeks) [23]	− 0.01 ( − 0.81, 0.79)		
		+ 10% (post 12 weeks) [23]	− 0.04 ( − 0.84, 0.76)		
	Hip extension			− 10% [24]	**0.71 (0.07, 1.35)**

SMD ± 95% CI are provided for each percentage increase or decrease in running step rate. SMD ± 95% CI presented in bold are statistically significant

Abbreviations: *ADDBREV* adductor brevis, *ADDLONG* adductor longus, *ADDMAG* adductor magnus, *GMAX* gluteus maximus, *GMED* gluteus medius, *GMIN* gluteus minimus, *IC* initial contact, *IR* internal rotation, *SART* sartorius, *TFL* tensor fasciae latae

**Table 10:** Pooled and single study results for kinetic, kinematic and muscle activation variables at trunk and pelvis

Variable		Preferred SR versus Increased SR		Preferred SR versus Reduced SR	
Kinetics	Peak sacral acceleration	+ 10% [44]	− 0.63 ( − 1.29, 0.02)	− 10% [44]	0.05 ( − 0.59, 0.68)
Kinematics	Average trunk flexion during stance phase	+ 10% [33, 34]	0.00 ( − 0.39, 0.39)		
	Average trunk flexion at IC	+ 10% [34]	− 0.01 ( − 0.51, 0.49)		
	Peak trunk flexion during stance phase	+ 10% [34]	0.05 ( − 0.45, 0.55)		
	Pelvic tilt			− 10% [24]	**1.40 (0.70, 2.10)**
	Peak contralateral pelvic drop	+ 10% (post 4 weeks) [23]	**1.39 (0.48, 2.30)**		
		+ 10% (post 12 weeks) [23]	**1.39 (0.48, 2.30)**		

SMD ± 95% CI are provided for each percentage increase or decrease in running step rate. SMD ± 95% CI presented in bold are statistically significant

Abbreviations: *IC* initial contact

**Table 11:** Segment coordination and coordination variability results from single studies

Variables		Preferred SR versus Increased SR	
Segment Coordination	Sagittal thigh versus sagittal shank: terminal swing in-phase	+ 10% [47]	− 0.11 ( − 0.98, 0.77)
	Sagittal thigh versus sagittal shank: terminal swing anti-phase	+ 10% [47]	** − 0.98 ( − 1.91, − 0.04)**
	Sagittal thigh versus sagittal shank: terminal swing distal segment	+ 10% [47]	0.11 ( − 0.77, 0.98)
	Sagittal thigh versus sagittal shank: terminal swing prox segment	+ 10% [47]	**0.98 (0.04, 1.91)**
	Sagittal thigh versus sagittal shank: early stance in-phase	+ 10% [47]	− 0.16 ( − 1.04, 0.72)
	Sagittal thigh versus sagittal shank: early stance anti-phase	+ 10% [47]	− 0.14 ( − 1.01, 0.74)
	Sagittal thigh versus sagittal shank: early stance proximal segment	+ 10% [47]	0.31 ( − 0.57, 1.19)
	Sagittal thigh versus sagittal shank: mid stance in-phase	+ 10% [47]	− 0.86 ( − 1.79, 0.06)
	Sagittal thigh versus sagittal shank: mid stance distal segment	+ 10% [47]	0.74 ( − 0.17, 1.65)
	Sagittal thigh versus sagittal shank: mid stance proximal segment	+ 10% [47]	− 0.36 ( − 1.24, 0.53)
	Sagittal thigh versus sagittal shank: late stance in-phase	+ 10% [47]	− 0.65 ( − 1.56, 0.25)
	Sagittal thigh versus sagittal shank: late stance distal segment	+ 10% [47]	0.46 ( − 0.43, 1.35)
	Sagittal thigh versus sagittal shank: late stance proximal segment	+ 10% [47]	0.32 ( − 0.56, 1.20)
	Sagittal thigh versus transverse shank: terminal swing in-phase	+ 10% [47]	− 0.11 ( − 0.99, 0.77)
	Sagittal thigh versus transverse shank: terminal swing anti-phase	+ 10% [47]	− 0.05 ( − 0.93, 0.82)
	Sagittal thigh versus transverse shank: terminal swing distal segment	+ 10% [47]	0.38 ( − 0.51, 1.27)
	Sagittal thigh versus transverse shank: terminal swing proximal segment	+ 10% [47]	− 0.62 ( − 1.52, 0.28)
	Sagittal thigh versus transverse shank: early stance in-phase	+ 10% [47]	− 0.32 ( − 1.20, 0.57)
	Sagittal thigh versus transverse shank: early stance anti-phase	+ 10% [47]	0.17 ( − 0.70, 1.05)
	Sagittal thigh versus transverse shank: early stance distal segment	+ 10% [47]	0.00 ( − 0.88, 0.88)
	Sagittal thigh versus transverse shank: early stance proximal segment	+ 10% [47]	0.35 ( − 0.53, 1.24)
	Sagittal thigh versus transverse shank: mid stance in-phase	+ 10% [47]	0.46 ( − 0.44, 1.35)
	Sagittal thigh versus transverse shank: mid stance anti-phase	+ 10% [47]	0.34 ( − 0.54, 1.23)
	Sagittal thigh versus transverse shank: mid stance distal segment	+ 10% [47]	− 0.48 ( − 1.37, 0.41)
	Sagittal thigh versus transverse shank: mid stance proximal segment	+ 10% [47]	0.20 ( − 0.68, 1.08)
	Sagittal thigh versus transverse shank: late stance in-phase	+ 10% [47]	0.19 ( − 1.07, 0.69)
	Sagittal thigh versus transverse shank: late stance anti-phase	+ 10% [47]	**1.01 (0.06, 1.95)**
	Sagittal thigh versus transverse shank: late stance distal segment	+ 10% [47]	− 0.47 ( − 1.36, 0.42)
	Sagittal thigh versus transverse shank: late stance proximal segment	+ 10% [47]	− 0.29 ( − 1.17, 0.59)
	Transverse thigh versus transverse shank: terminal swing in-phase	+ 10% [47]	− 0.32 ( − 1.20, 0.57)
	Transverse thigh versus transverse shank: terminal swing anti-phase	+ 10% [47]	− 0.31 ( − 1.19, 0.58)
	Transverse thigh versus transverse shank: terminal swing distal segment	+ 10% [47]	0.53 ( − 0.37, 1.43)
	Transverse thigh versus transverse shank: terminal swing proximal segment	+ 10% [47]	− 0.04 ( − 0.91, 0.84)
	Transverse thigh versus transverse shank: early stance in-phase	+ 10% [47]	− 0.31 ( − 1.19, 0.58)
	Transverse thigh versus transverse shank: early stance anti-phase	+ 10% [47]	0.31 ( − 0.58, 1.19)
	Transverse thigh versus transverse shank: early stance distal segment	+ 10% [47]	− 0.06 ( − 0.93, 0.82)
	Transverse thigh versus transverse shank: early stance proximal segment	+ 10% [47]	0.00 ( − 0.88, 0.88)
	Transverse thigh versus transverse shank: mid stance in-phase	+ 10% [47]	− 0.63 ( − 1.54, 0.27)
	Transverse thigh versus transverse shank: mid stance anti-phase	+ 10% [47]	0.00 ( − 0.88, 0.88)
	Transverse thigh versus transverse shank: mid stance distal segment	+ 10% [47]	0.12 ( − 0.75, 1.00)
	Transverse thigh versus transverse shank: mid stance proximal segment	+ 10% [47]	0.39 ( − 0.50, 1.27)
	Transverse thigh versus transverse shank: late stance in-phase	+ 10% [47]	0.10 ( − 0.78, 0.97)
	Transverse thigh versus transverse shank: late stance anti-phase	+ 10% [47]	0.03 ( − 0.84, 0.91)
	Transverse thigh versus transverse shank: late stance distal segment	+ 10% [47]	− 0.57 (1.47, 0.32)
	Transverse thigh versus transverse shank: late stance proximal segment	+ 10% [47]	0.33 ( − 0.55, 1.22)
	Transverse shank versus frontal rearfoot: terminal swing in-phase	+ 10% [47]	0.00 ( − 0.88, 0.88)
	Transverse shank versus frontal rearfoot: terminal swing anti-phase	+ 10% [47]	− 0.31 ( − 1.20, 0.57)
	Transverse shank versus frontal rearfoot: terminal swing distal segment	+ 10% [47]	0.09 ( − 0.79, 0.96)
	Transverse shank versus frontal rearfoot: terminal swing proximal segment	+ 10% [47]	0.21 ( − 0.66, 1.09)
	Transverse shank versus frontal rearfoot: early stance in-phase	+ 10% [47]	0.09 ( − 0.79, 0.96)
	Transverse shank versus frontal rearfoot: early stance anti-phase	+ 10% [47]	− 0.08 ( − 0.95, 0.80)
	Transverse shank versus frontal rearfoot: early stance distal segment	+ 10% [47]	− 0.10 ( − 0.97, 0.78)
	Transverse shank versus frontal rearfoot: early stance proximal segment	+ 10% [47]	0.18 ( − 0.70, 1.06)
	Transverse shank versus frontal rearfoot: mid stance in-phase	+ 10% [47]	0.39 ( − 0.50, 1.28)
	Transverse shank versus frontal rearfoot: mid stance anti-phase	+ 10% [47]	− 0.20 ( − 1.08, 0.68)
	Transverse shank versus frontal rearfoot: mid stance distal segment	+ 10% [47]	− 0.66 ( − 1.56, 0.25)
	Transverse shank versus frontal rearfoot: mid stance proximal segment	+ 10% [47]	0.00 ( − 0.88, 0.88)
	Transverse shank versus frontal rearfoot: late stance in-phase	+ 10% [47]	0.40 ( − 0.49, 1.29)
	Transverse shank versus frontal rearfoot: late stance distal segment	+ 10% [47]	− 0.40 ( − 1.28, 0.49)
	Transverse shank versus frontal rearfoot: late stance proximal segment	+ 10% [47]	− 0.11 ( − 0.99, 0.77)
Coordination Variability	Sagittal thigh versus sagittal shank: terminal swing	+ 10% [47]	0.49 ( − 0.41, 1.38)
	Sagittal thigh versus sagittal shank: early stance	+ 10% [47]	− 0.33 ( − 1.22, 0.55)
	Sagittal thigh versus sagittal shank: mid stance	+ 10% [47]	− 0.17 ( − 1.05, 0.71)
	Sagittal thigh versus sagittal shank: late stance	+ 10% [47]	0.41 ( − 0.48, 1.30)
	Sagittal thigh versus transverse shank: terminal swing	+ 10% [47]	− 0.11 ( − 0.99, 0.77)
	Sagittal thigh versus transverse shank: early stance	+ 10% [47]	**1.70 (0.64, 2.75)**
	Sagittal thigh versus transverse shank: mid stance	+ 10% [47]	0.80 ( − 0.12, 1.71)
	Sagittal thigh versus transverse shank: late stance	+ 10% [47]	0.74 ( − 0.17, 1.65)
	Transverse thigh versus transverse shank: terminal swing	+ 10% [47]	− 0.08 ( − 0.95, 0.80)
	Transverse thigh versus transverse shank: early stance	+ 10% [47]	**1.18 (0.22, 2.15)**
	Transverse thigh versus transverse shank: mid stance	+ 10% [47]	0.49 ( − 0.40, 1.39)
	Transverse thigh versus transverse shank: late stance	+ 10% [47]	− 0.02 ( − 0.09, 0.85)
	Transverse shank versus frontal forefoot: terminal swing	+ 10% [47]	**1.33 (0.34, 2.32)**
	Transverse shank versus frontal forefoot: early stance	+ 10% [47]	0.44 ( − 0.45, 1.33)
	Transverse shank versus frontal forefoot: mid stance	+ 10% [47]	− 0.02 ( − 0.90, 0.86)
	Transverse shank versus frontal forefoot: late stance	+ 10% [47]	− 0.20 ( −1.08, 0.68)

SMD ± 95% CI are provided for each percentage increase or decrease in running step rate. SMD ± 95% CI presented in bold are statistically significant

#### Biomechanics

Twenty-two studies [10, 12–14, 18–25, 28, 30, 32, 33, 35, 37–39, 42, 45] were identified evaluating biomechanical differences between running with a preferred step rate and an increased step rate, and 13 studies [12–14, 19, 20, 24, 25, 28, 32, 33, 37, 39, 42] were identified evaluating biomechanical differences between running with a preferred step rate and a reduced step rate. A total of 221 variables were evaluated (Tables 5, 6, 7, 8, 9,10, 11).

##### Spatiotemporal Gait Parameters

Nine studies [24, 26, 30, 33, 44, 45, 49, 54, 57] were identified evaluating running spatiotemporal gait parameters. Eight studies [26, 30, 33, 44, 45, 49, 54, 57] evaluated differences in gait parameters between running with a preferred step rate and an increased step rate, while seven studies [24, 26, 30, 44, 45, 49, 54] evaluated differences between running with a preferred step rate and a reduced step rate.

**Step length:** In recreational runners, compared to running with a preferred step rate: moderate evidence indicated a shorter *step length* with a *10% increase in step rate* (2HQ [30, 33] and 2MQ [44, 49]; 0.93, 0.49 to 1.37; *I*^2^ = 52%); and moderate evidence indicated a longer *step length* with a *10% reduction in step rate* (1HQ [30], 2MQ [44, 49] and 1LQ [24]; − 0.76, − 1.31 to − 0.21; *I*^2^ = 70%).

**Contact time:** In recreational runners, compared to running with a preferred step rate: limited evidence indicated no difference in *contact time* with a *10% increase in step rate* (1HQ [30] and 1MQ [45]; 0.50, -0.02 to 1.03; *I*^2^ = 0%); and limited evidence indicated an increase in *contact time* with a *10% reduction in step rate* (1HQ [30] and 1MQ [45]; − 0.95, − 1.49 to − 0.40; *I*^2^ = 0%).

##### Ground Reaction Forces, Loading Rates and Braking Impulse

Ten studies [25, 30, 31, 33, 38, 41, 44, 49, 50, 54] were identified evaluating ground reaction force and loading rate variables. All studies evaluated biomechanical differences between running with a preferred step rate and an increased step rate, while six studies [25, 30, 31, 44, 49, 54] evaluated biomechanical differences between running with a preferred step rate and a reduced step rate.

**Ground reaction forces:** In recreational runners, *increasing step rate by 10%* was associated with limited evidence of no difference in *peak vertical ground reaction force* (1HQ [33] and 1MQ [49]; 0.24, -0.11 to 0.59; *I*^2^ = 0%).

**Loading rates:** In recreational runners, *increasing running step rate by 10%* was associated with no difference in *average vertical loading rate* (1HQ [41] and 1MQ [50]; 0.24, − 0.23 to 0.70; *I*^2^ = 0%) and *vertical instantaneous loading rate* (1HQ [41] and 1MQ [50]; − 0.04, − 0.50 to 0.42; *I*^2^ = 0%).

**Braking impulse:** In recreational runners, *reducing step rate by 10%* was associated with limited evidence of increased *braking impulse* (1HQ [30] and 1MQ [49]; − 0.73, − 1.08 to − 0.37; *I*^2^ = 0%).

##### Foot, Ankle, and Lower Leg

Nineteen studies [22, 26, 30–36, 38, 39, 41, 44, 45, 47, 49, 50, 54, 57] evaluated 81 biomechanical variables at the foot, ankle, and lower leg. All studies evaluated biomechanical differences between running with a preferred step rate and an increased step rate, while ten studies [26, 30, 31, 35, 36, 39, 44, 45, 49, 54] also evaluated biomechanical differences between running with a preferred step rate and a reduced step rate.

**Kinetics:** In recreational runners, *increasing step rate by 10%* was associated with moderate evidence of no difference in *peak tibial acceleration* (2HQ [31, 41] and 2MQ [44, 50]; 0.06, − 0.29 to 0.42; *I*^2^ = 8%); and limited evidence of no difference in *negative ankle work* (2 MQ [44, 49]; − 0.01, − 0.36 to 0.33; *I*^2^ = 0%). *Increasing step rate by 5%* was associated with moderate evidence of no difference in *rearfoot peak pressure* (2HQ; 0.18, − 0.15 to 0.51; *I*^2^ = 0%) and *rearfoot contact time* (2HQ; − 0.07, − 0.41 to 0.26; *I*^2^ = 0%).

In recreational runners, *reducing step rate by 10%* was associated with limited evidence of increased *negative ankle work* (2 MQ [44, 49];−  0.38, − 0.73 to − 0.03; *I*^2^ = 0%) and no difference in *peak tibial acceleration* (1HQ [31] and 1 MQ [44]; − 0.42, − 0.93 to 0.08; *I*^2^ = 0%). *Reducing step rate by 5%* was associated with moderate evidence of no difference in *rearfoot peak pressure* (2HQ [35, 39]; − 0.14, − 0.48 to 0.19; *I*^2^ = 0%), *rearfoot max force* (2HQ; − 0.14, − 0.47 to 0.19; *I*^2^ = 0%), and *rearfoot contact time* (2HQ [35, 39]; − 0.23, − 0.56 to 0.10; *I*^2^ = 0%).

**Kinematics:** In recreational runners, *increasing step rate by 10%* was associated with moderate evidence of reduced *foot strike angle* (2HQ [22, 33] and 1MQ [49]; 0.62, 0.34 to 0.09; *I*^2^ = 0%); and limited evidence of no difference in *average plantar/dorsiflexion* at initial contact (1HQ [34] and 1MQ [45]; 0.23, − 0.20 to 0.67; *I*^2^ = 0%). *Increasing step rate by 5%* was associated with limited evidence of reduced *foot strike angle* (1HQ [22] and 1MQ [49]; 0.39, 0.09 to 0.69; *I*^2^ = 0%).

##### Knee

Fourteen studies [23, 26, 29, 30, 32–34, 36, 38, 44, 45, 47, 49, 51, 55] evaluated 64 biomechanical variables at the knee. All studies evaluated biomechanical differences between running with a preferred step rate and an increased step rate, while seven studies [26, 30, 36, 44, 45, 49, 51] also evaluated biomechanical differences between running with a preferred step rate and a reduced step rate.

**Kinetics:** In recreational runners, *increasing step rate by 10%* was associated with moderate evidence of reduced *peak knee extensor moment* (2HQ [29, 33] and 1MQ [49]; 0.50, 0.18 to 0.81; *I*^2^ = 0%); and limited evidence of reduced *peak patellofemoral joint stress* (2HQ [29, 33]; 0.56, 0.07 to 1.05; *I*^2^ = 0%) and reduced *negative knee work* (2 MQ [44, 49]; 0.84, 1.20 to 0.48; *I*^2^ = 0%). In recreational runners, *reducing step rate by 10%* was associated with limited evidence of reduced *negative knee work* (2 MQ [44, 49]; 0.88, 0.52 to 1.25; *I*^2^ = 0%).

**Kinematics:** In recreational runners, *increasing step rate by 10%* was associated with strong evidence of reduced *peak knee flexion angle* (3HQ [29, 33, 34] and 2MQ [47, 49]; 0.66, 0.40 to 0.92; *I*^2^ = 0%); and moderate evidence of no difference in *average knee flexion at initial contact* (1HQ [34] and 2MQ [45, 49]; − 0.23, − 0.53 to 0.07; *I*^2^ = 0%). *Increasing step rate by 5%* was associated with limited evidence of no difference in *average knee flexion at initial contact* (2 MQ [45, 49]; − 0.19, − 0.57 to 0.18; *I*^2^ = 0%).

In recreational runners, *reducing step rate by 10%* was associated with limited evidence of no difference in *average knee flexion at initial contact* (2 MQ [45, 49]; 0.18, − 0.20 to 0.55; *I*^2^ = 0%). *Reducing step rate by 5%* was associated with limited evidence of no difference in *average knee flexion at initial contact* (2 MQ [45, 49]; 0.15, − 0.22 to 0.53; *I*^2^ = 0%).

##### Hip

Thirteen studies [23, 24, 26, 32–34, 36, 38, 41, 44, 45, 49, 51, 55] evaluated 67 biomechanical variables at the hip. Twelve studies [23, 26, 32–34, 36, 38, 41, 44, 45, 49, 51, 55] evaluated biomechanical differences between running with a preferred step rate and an increased step rate, while seven studies [24, 26, 36, 44, 45, 49, 51] evaluated biomechanical differences between running with a preferred step rate and a reduced step rate.

**Kinetics:** In recreational runners, *increasing step rate by 10%* was associated with limited evidence of reduced *negative hip work* (2 MQ [44, 49]; 0.55, 0.91 to 0.20; *I*^2^ = 0%). In recreational runners, *reducing step rate by 10%* was associated with limited evidence of increased *negative hip work* (2 MQ [44, 49]; − 0.67, − 1.02 to − 0.31; *I*^2^ = 0%).

**Kinematics:** In recreational runners, *increasing step rate by 10%* was associated with moderate evidence of reduced *peak hip adduction* during stance phase (2HQ [34, 41] and 1MQ [49]; 0.40, 0.11 to 0.69; *I*^2^ = 0%); and limited evidence of reduced *peak hip flexion* during stance phase (1HQ [34] and 1MQ [49]; 0.42, 0.10 to 0.75; *I*^2^ = 0%), no difference in *average hip flexion* at initial contact (1HQ [34] and 1MQ [45]; 0.14, − 0.29 to 0.57; *I*^2^ = 0%) and no difference in *peak hip internal rotation* during stance phase (1HQ [34] and 1MQ [49]; 0.07, − 0.25 to 0.38; *I*^2^ = 0%).

##### Trunk and Pelvis

Five studies [23, 24, 33, 34, 44] evaluated five biomechanical variables at the trunk and pelvis (Table 10). Four studies [23, 33, 34, 44] evaluated biomechanical differences between running with a preferred step rate and an increased step rate, while two studies [24, 44] evaluated biomechanical differences between running with a preferred step rate and a reduced step rate.

**Kinetics:** No data pooling was possible for any trunk or pelvis kinetic findings.

**Kinematics:** In recreational runners, *increasing step rate by 10%* was associated with moderate evidence of no difference in *average trunk flexion* during stance phase (2 HQ [33, 34]; 0.00, − 0.39 to 0.39; *I*^2^ = 0%).

## Discussion

This systematic review summarises the literature and provides a meta-analysis to estimate the effects of changing running step rate on injury, performance and biomechanics. Findings indicate there is insufficient evidence to conclusively determine the effects of altering running step rate on injury and performance. However, a large body of biomechanical research that can guide clinical practice and future research was identified. Our meta-analysis found that increasing running step rate generally results in a reduction (or no change) in kinetic, kinematic, and loading rate variables at the ankle, knee, and hip. In contrast, reducing running step rate generally resulted in an increase (or no change) in kinetic, kinematic, and loading rate variables.

### Injury

Despite coaches and clinicians commonly increasing running step rate in the management of running injuries [8], only two studies [23, 55] have evaluated the effect of this practice on clinical outcomes in injured runners. These studies indicate that increasing preferred running step rate by 7.5% (mean baseline preferred step rate: 163 per minute) [55] and 10% (mean baseline preferred step rate: 166 per minute) [23] is associated with improved pain and function in runners with patellofemoral pain at 4 weeks [23], 6 weeks [55], and 3 months [23]. Although these findings are promising, neither study used a control or comparator group, limiting the ability to evaluate efficacy. With this in mind, it is worth noting that a clinical trial, not included in this review due to using a combined running retraining strategy, found that increasing step rate by 7.5% to 10% in conjunction with other retraining strategies (instruction to run softer and adopt a non-rearfoot strike pattern if deemed necessary) did not provide additional benefits in runners with patellofemoral symptoms compared to education about symptom management and training modification [15]. Considering these findings, and those from the two case-series studies included in this review, high-quality clinical trials are required to establish the efficacy of increasing running step rate for the management of patellofemoral pain, and other common running-related injuries.

### Performance

This review found insufficient evidence to determine the effect of changing running step rate on performance. Five studies focussed on surrogate measures of performance inclusive of VO_2_ [42, 53], RPE [49], metabolic cost [26], awkwardness [50], and effort [50]. Although findings from these studies indicated that increasing step rate may have a detrimental effect on some subjective measures of performance (e.g. RPE, effort and a feeling of awkwardness), there was no evidence to indicate a detrimental effect on physiological measures of running performance (e.g. VO_2_). Of note, very limited evidence from a recent cross-sectional study found that changing a runner’s preferred step rate results in an increase in metabolic energy consumption, proposed to result from large increases in positive ankle power when decreasing step rate, and large increases in positive hip power when increasing step rate [26, 51]. The studies included in this review relate to the immediate effect of changing step rate on performance, and as such the long-term effect of a change in step rate after a period of habituation remains unknown.

### Biomechanics

The findings from this review provide some biomechanical rationale for increasing running step rate to reduce numerous kinetic, kinematic, and loading rate variables at the ankle, knee and hip, while also resulting in changes to spatiotemporal measures.

As expected, pooled data provide moderate evidence that increasing and decreasing running step rate by 10% results in a shorter and longer step length, respectively. Additionally, limited evidence indicated an increase in contact time when step rate is reduced by 10%. However, limited evidence indicated that a 10% increase in step rate provides no effect on contact time. Single studies (not included in meta-analysis) provide very limited evidence that contact time decreased with a 15% and 30% increase in step rate, but this was not observed with smaller increases in step rate (5% and 8%). While shorter contact time is associated with faster running speeds, the effect on performance is not known [59, 60]. Further, very limited evidence indicated a reduction in COM to heel distance with a 10% increase in step rate, which is consistent with the finding that a shorter step length is associated with an increase in step rate. Although changing running step rate has been shown to provide effects on spatiotemporal measures, any clinical benefits from these changes remain unknown as there is a lack of evidence linking spatiotemporal gait parameters to running injuries [61].

The relationship between vertical ground reaction forces and running-related injury has been extensively researched, with vertical loading rate reported to have the most consistent association with injury [62–64]. Pooled data from this review provide limited evidence that increasing step rate does not change peak vertical ground reaction force, average vertical loading rate, and vertical instantaneous loading rate [1, 5, 10, 11]. These findings were consistent across multiple single studies and included step rate increases from 5 to 30%. In contrast, however, a single study found that in-field gait retraining (8 sessions in 4 weeks to increase running step rate by 7.5%) in runners with high impact forces reduced average vertical load rate and vertical instantaneous load rate [40]. A possible explanation for this finding, compared to other studies, is they included a targeted population of runners with high impact loads (≥ 85 body weights/second in either limb). Limited evidence from single studies indicates an increase in vertical ground reaction force, average vertical loading rate, and vertical instantaneous loading rate with a 30% reduction in step rate [25, 54]. However, this finding was not observed with smaller reductions in step rate (5% to 15%). We found limited evidence that a reduction in braking impulse is associated with a 10% increase in step rate [30, 49]. Peak braking force is an impact variable likely to be of interest to runners as it been identified as a predictor of running-related injuries [65]. It would therefore be beneficial if further studies could confirm if braking impulse can be reduced by increasing step rate, and ideally explore if this reduces injury risk in runners.

At the foot and ankle, limited evidence indicated a reduction in negative ankle work with a reduced step rate, and moderate evidence identified a reduction in foot strike angle with an increase in step rate. This latter finding is likely to be of interest to coaches and runners as reducing foot strike angle, or converting to a non-rearfoot strike pattern, are other commonly used running retraining strategies [8]. The findings of this review indicate that increasing running step rate may be a relatively safe running retraining strategy if attempting to reduce foot strike angle, as it achieves this goal while providing an overall reduction in kinetic, kinematic and loading rate variables. All other biomechanical variables included in this review indicate no effect at the foot and ankle with a change in step rate.

The biomechanical effects observed at the knee with an increase in step rate provide rationale for potential clinical benefits of running-related knee injuries, such as patellofemoral pain. An increase in running step rate was associated with strong evidence of a reduction in peak knee flexion angle [29, 33, 34, 47, 49], moderate evidence of a reduction in patellofemoral joint stress [29, 33] and peak knee extensor moment [29, 33, 49], and limited evidence of a decrease in negative knee work [44, 49]. Two studies that reported a reduction in patellofemoral joint stress and peak knee extensor moment with an increase in step rate made these observations in runners with patellofemoral pain [29, 33]. It is biologically plausible that reducing patellofemoral joint stress and peak knee extensor moments at the site of injured tissue is likely to provide benefits in pain and function. Combined with the clinical benefits reported in case-series studies of increasing step rate in runners with patellofemoral pain, these biomechanical findings justify the need for clinical trials to establish efficacy of increasing step rate in runners with patellofemoral pain.

At the hip, moderate evidence indicated a reduction in peak hip adduction during stance phase with a 10% increase in step rate. As greater peak hip adduction during running has previously been associated with common running injuries inclusive of patellofemoral pain, ITB friction syndrome and gluteal tendinopathy [66], it could be hypothesised that increasing step rate could be clinically beneficial in the management of these injuries. Of interest, a reduction in peak hip adduction was also observed at 4 weeks and 12 weeks post gait retraining to increase running step rate by 10% [23], indicating that changes can be maintained over time. Limited evidence indicated an increase in step rate reduces both hip flexion during stance and negative hip work, with the latter finding being of particularly interest given that reducing negative hip work has been theorised to be beneficial in the management of running injuries, due to its association with improved lower limb alignment at initial contact [49].

At the trunk and pelvis, no data were able to be pooled and most findings indicate that changing running step rate does not change biomechanical variables. The exceptions were very limited evidence from single studies indicating reduced pelvic tilt immediately, and reduced contralateral pelvic drop at 4 weeks and 12 weeks post, an increase in running step rate by 10% [23].

Our review found that many biomechanical variables can be altered by instructing a runner to increase or decrease their preferred step rate, but it is difficult to determine if the biomechanical variations occur to achieve the goal of a change in step rate or are a result of a change. Therefore, the biomechanical findings of this review reflect what occurs in clinical practice, whether a mechanism or outcome, when runners are instructed to change their step rate.

### Clinical Implications

Insufficient evidence exists to determine the effects of increasing running step rate on injury and performance. Therefore, the rationale for its use largely relies on the knowledge that numerous biomechanical variables can be changed with each step, as found in this review. At present, there is no evidence to guide clinicians in identifying runners most likely to benefit from an increase in running step rate. Clinicians will therefore need to determine its appropriateness based on each runner’s clinical presentation, short- and long-term running goals, and a runner’s desire to change their running gait.

It is also noteworthy that the studies included in this systematic review predominantly included recreational runners, and consideration must therefore be given to the potential differences in response among elite athletes.

If an increase in running step rate is adopted by an injured runner, any reduction in biomechanical load at the site of injury could help to reduce pain, and potentially maintain running load. Increasing step rate may only be required in the short-term, allowing for a continuation of running while the injury is rehabilitated. The runner may then be able to return to their preferred step rate once the injury is resolved. A long-term change in a runner’s preferred step rate may be warranted where a chronic running-related injury is being managed, or where the runner’s preferred step rate is considered by the clinician as being a factor for ongoing injury risk [8]. It is worth noting that multiple single studies looking at increasing step rate as a running retraining intervention found that increases in step rate were maintained across time frames from 12 to 12 weeks [23, 27, 40].

Consideration must also be given to baseline step rate before determining the appropriateness of implementing a change in step rate. Mean baseline values for step rate reported in studies within this review range from 160 [56] to 172 steps per minute [33], with an increase in reported step rate values as high as 192 steps per minute with a 15% increase [19]. It is likely that the observed effects that occur when a runner changes their step rate are likely to be dependent on each runner’s preferred step rate, which was not explored by any study included in this review.

Clinicians, coaches, and runners need to be mindful that any observed reduction in kinetic, kinematic or loading rate variables per step may be off-set by the increased number of steps taken per minute of running (up to 30% in some studies)—possibly leading to an equal or greater accumulation of loading over a set distance or time. Such consideration is important, as most running-related injuries are proposed to result from an accumulation of tissue load, rather than just the magnitude of each application of load. Of interest, one study has investigated the effects of running with a shortened step length (i.e. increased step rate) on patellofemoral kinetics with each step and over a set distance, finding that patellofemoral kinetics decreased by 15 to 20% with each step and decreased by 9 to 12% per kilometre [18]. Despite these promising findings, given the uncertainty regarding other biomechanical variables, when runners are increasing their running step rate, a transition period may be necessary to allow adaption to any new tissue loads experienced with the change in running gait.

### Limitations and Future Directions

The findings of this review need to be considered in the context of five key limitations. First, there is limited research on the effects of changes in step rate on injury and performance, which are likely to be the main motivators for changing running step rate among runners, clinicians and coaches. Second, as most studies included in this review investigated the immediate effects of changes in step rate, the longer-term effects remain largely unknown. Third, participants used in most studies were healthy (i.e. uninjured) and relatively young so it remains unclear if the biomechanical and performance effects may differ among injured and / or older runners. Fourth, we excluded studies that combined interventions with changes in running step rate. Importantly, changes in step rate may be accompanied by other running retraining strategies (e.g. change in footstrike) or interventions (e.g. change in footwear) in research and practice. Therefore, our findings may only apply in cases where changing step rate is the sole intervention. Fifth, data were not able to be extracted from some studies and were not provided upon request, which may have led to the omission of potentially relevant data in our results. Finally, we recognise that the association between injury and some of the biomechanical variables included in this review have not been fully established. In consideration of these shortcomings, it would be beneficial for future studies to investigate the immediate and longer-term effects of altered running step rate on biomechanical and performance variables known to, or proposed to be, associated with injury, or actual patient-focused outcomes and running performance.

## Conclusion

This systematic review highlights that increasing running step rate will, in general, either provide no change or reduce kinetic, kinematic and loading rate variables at the ankle, knee and hip—all common injury sites in runners. In contrast, no change or an increase in kinetic, kinematic and loading rate variables were generally observed when running step rate was reduced. At present there is insufficient evidence to conclusively determine the effects of altering running step rate on injury or performance. While research relating to the effect of changing running step rate on injury and performance appears to be scarce, it does suggest that increasing running step rate could be effective in reducing load through targeted tissues and therefore appropriate in certain injury presentations, such as patellofemoral pain. It also suggests that while increasing running step rate may not improve performance, if utilised as an intervention in the management of an injury, it is unlikely to have a detrimental effect on performance.

## Data Availability

All data are provided within the main manuscript and supplementary files.
